# Healthy ageing for older adult people with intellectual disability: a scoping review

**DOI:** 10.1186/s13690-025-01528-0

**Published:** 2025-02-27

**Authors:** Nadia El Mrayyan, Marianne Holmgren, Gerd Ahlström

**Affiliations:** 1 Department of Health Sciences, Faculty of Medicine, Lund University, P.O., Box 117, Lund, 221 00 Sweden; 2 Department of Public Health, College of Health Science, Abu Dhabi University, Abu Dhabi, United Arab Emirates

**Keywords:** Healthy ageing, Successful ageing, Active ageing, Ageing in place, Scoping review, Older adults, Intellectual disability, Developmental disability, Intervention studies, Evidence-based practice

## Abstract

**Background:**

The increasing longevity of people with intellectual disability creates a need for a healthy-ageing perspective, translated into evidence-based interventions in this multi-morbidity group. Accordingly, the aim of this scoping review was to identify, summarise and analyse the empirical research on healthy ageing in older adults with intellectual disability.

**Methods:**

This review was based on the PRISMA 2020 guidelines for Scoping Reviews (PRISMA-ScR) and a PICO protocol (Patient/population, Intervention, Comparison/control, and Outcome). Empirical studies in English were included if they concerned older adults with intellectual disability with an average age of at least 45 and were based on a clearly expressed healthy-ageing perspective. An information specialist conducted a search in 11 databases with no geographical or temporal restrictions. Two independent researchers performed study selection, quality assessment and data extraction. Disagreements were resolved in consultation with a third researcher. A textual narrative synthesis was based on PICO domains and the seven research questions.

**Results:**

The 11 studies were all from developed countries and had different designs: qualitative, mixed-method and one systematic review. Only three studies highlighted the term “healthy ageing”, most used synonymous terms. Eight studies focused on healthy ageing on the individual level, three on the organisational and societal level. The intervention studies in the systematic review were mainly nonrandomised, concerned interventions varying in intensity and duration, considered different research questions and employed different outcome measures.

**Conclusions:**

The findings highlight a major knowledge gap concerning evidence-based interventions with a healthy-ageing perspective in the case of older adults with intellectual disability. There is an urgent need to initiate healthy-ageing studies in developing countries, where such people are even more vulnerable to stigma and discrimination than those in developed countries. Our findings confirm the need to scale up healthy-ageing interventions in line with the WHO’s ambition to develop evidence-based approaches to optimise the functional capacity of all older people, including older adults with intellectual disability, by 2030.

**Registration:**

The study is registered in the International Prospective Register of Systematic Reviews (PROSPERO), CRD42022337211 (13 June 2022).

**Supplementary Information:**

The online version contains supplementary material available at 10.1186/s13690-025-01528-0.

**Table:** 

Text box 1. Contributions to the literature
• The study highlights how little research there has been on healthy ageing from a public health perspective, also the variation in the terms used in such research
• The identified knowledge gap in the literature is specific to healthy-ageing interventions for older people with intellectual disability
• Intellectual disability from an early age makes people more vulnerable to ageing, highlighting the need for healthy-ageing interventions
• Primary care and disability services must work in partnership to meet the ageing-related needs of the growing population worldwide
• There is a need to increase public involvement and participatory research practices to improve healthy-ageing strategies

## Background

Ageing with intellectual disability (ID) is usually a more complex process than is normal in the general population, involving conditions such as co-morbidities and age-related health conditions earlier in life which progress over time [1–4]. Epidemiological research in recent decades has confirmed that older adults with ID have poorer mental and physical health than people without such disability [5–11]. Longevity in the group of people with ID has increased at least at the same pace as in the general population [12–14]. However, the gap in longevity for people with ID as compared with the general population is still about 20 years in developed countries and even larger in developing countries [3, 15–17]. These factors show how significantly more vulnerable older adults with ID are than others [18, 19] thereby showing the need for healthy-ageing interventions in their case.

This need for healthy-ageing interventions increases as the people with ID get older and thus live with long-term health conditions. The interventions prevent unnecessary illness and hospital care, hence increasing people’s well-being and reducing society’s costs [20, 21]. Such interventions are in alignment with the WHO conceptualisation of healthy ageing in terms of “the process of developing and maintaining the functional ability that enables wellbeing in older age” [22, 23], applied in the comprehensive “global strategy and action plan on ageing and health“ [24], and in the vision of implementation of the first action plan “Decade of Healthy Ageing” 2021–2030 [25]. However, good health in older age is not equally distributed, either between or within populations [24, 26], which is in contravention of the United Nations Convention on the Rights of Persons with Disabilities [27]. A crucial consequence of the cumulative impact of social and economic determinants of health in different groups in older age is that the people with the greatest health needs tend to also be those with the least access to the resources that might foster health [24, 26].

A first step in line with the global policy of implementing evidence-based action on ageing and health [24] for older adults with ID is to explore and analyse the existing knowledge in this area. Therefore the aim of this scoping review has been to identify, summarise and analyse the existing empirical research on healthy ageing in older adults with ID. The following research questions specify this aim:

1. What is the definition of the term “healthy ageing” in the literature concerning older adults with intellectual disability?
2. What terms similar to “healthy ageing” are used in the literature?
3. How is healthy ageing promoted through research concerning older adults with intellectual disability on the individual, the organisational and the societal level?
4. What are the similarities and differences in the description of healthy ageing between developing and developed countries and continents?
5. What part do participatory research and public involvement play in the healthy-ageing research concerning older adults with intellectual disability?
6. Are ethical issues or dilemmas taken up in connection with healthy ageing?
7. What are the knowledge gaps within healthy-ageing research and interventions for older adults with intellectual disability?

## Methods

Scoping reviews are a type of knowledge synthesis, using a systematic approach to map evidence on a topic and identify main concepts, theories, sources and knowledge gaps [28]. This review applied systematic methods following PRISMA 2020 (Preferred Reporting Items for Systematic reviews and Meta-Analyses), [29]. The PRISMA 2020 is relevant for mixed-methods systematic reviews, covering both quantitative and qualitative studies, and was completed with relevant guideline such as PRISMA for Scoping Reviews (PRISMA-ScR), [30] (see Supplementary Material 1). The study is registered in the International Prospective Register of Systematic Reviews (PROSPERO), CRD42022337211 (13 June 2022), available from https://www.crd.york.ac.uk/prospero/#searchadvanced).

### Eligibility criteria

A PICO (Population, Intervention/issue of interest, Comparison, Outcome) protocol was developed to determine which studies to include [31]. The language criterion was limited to English and the search terms were based on the research questions and the PICO protocol. A pilot study was carried out by the information specialist (MB); and after discussion with the first and third authors, two changes were made. The age was changed from 50 years and older to 45 years and older or a mean age of 45 which was based on how the age range appeared in some databases. This is in line with previous research reporting that in people with ID the ageing process starts earlier than in the general population [12]. The pilot study resulted in 1307 hits but we strove to get as much coverage as possible, therefore the main literature search was extended to include the search terms “policy”, “guidelines”, “legislation” and “ageing in place” in combination with “healthy ageing”.

When it comes to the main literature search, the terms and dates of coverage for each database are specified in Supplementary Material 2. MeSH terms were applied, modified in accordance with the vocabulary of the particular database. The following main MeSH terms (with “and/or”) were used in different combinations and with synonymous terms or different spelling (see Supplementary Material 2 for further details): 1) Intellectual disability, Down’s Syndrome, mental retardation, developmental disability, learning disability; 2) aged, adult, older, middle-aged, ageing, elderly, old age, senior, older adult, over 45, geriatric; 3) healthy ageing, ageing well, successful ageing, active ageing, healthy living, health indicator, health promotion, healthy, prevention, intervention, promotion (see details in Table 1. and Supplementary Material 2).

**Table 1:** Inclusion and exclusion criteria according to PICO used for studies about healthy ageing in older adult people with intellectual disability (ID)

**Patients/Population**
**Study eligible for inclusion if any of these criteria regarding participants were met:** 1. Older adults with intellectual disability (ID) aged 45 years or older (half or more of the participants or 45 years as mean age). Includes all sub-diagnoses of ID (for example Down’s syndrome, Retts syndrome) and autism if they also have ID. 2. Relatives of, or staff or politicians concerned with, older adults with (ID) aged 45 years or older (half or more of the participants or 45 years as mean age). 3. Older adults (aged 45 years or older)) with other diagnoses or disabilities but where the primary focus of the study is people in the same age-range with ID.
**Interventions/programmes**
**Eligible for inclusion were any of the following studies:** 1. Studies of healthy-ageing interventions implementation for older adults with ID within different settings. 2. Studies of the prevention of ethical dilemmas in healthy-ageing interventions for older adults with ID. 3. Literature reviews, scoping and systematic reviews of healthy ageing or subjects relevant to healthy ageing. 4. Qualitative studies as a part of the evaluation of interventions or exploring theoretical concepts concerning healthy ageing.
**Comparison/control groups**
**For the sake of comparison the following were eligible for inclusion:** 1. Studies of healthy-ageing interventions in developed and developing countries. 2. Cross-sectional surveys of different age groups with a purpose relevant to healthy ageing.
**Outcome measures**
**Studies that addressed the research questions were eligible for inclusion:** 1. Healthy-ageing interventions in developed or/and developing countries. 2. Definitions and theories (theoretical content) of healthy ageing for older adults with ID or synonymous concepts. 3. Participatory research within healthy ageing interventions for older adults with ID. 4. Ethical questions and dilemmas.
**Exclusion criteria**
1. People less than 45 years old. 2. Languages other than English. 3. Duplicated publications. 4. Other cognitive disabilities than ID. 5. Qualitative studies about experiences of people with ID, or experiences of relatives, staff or politicians, without any distinct connection to healthy ageing. 6. Epidemiological studies about diseases, illnesses or health risk factors (sleep difficulties, obesity etc.). 7. Preclinical medical trials. 8. Solely preclinical studies (for example gene research) or with basic psychological focus (for example testing of memory in people with ID). 9. Study protocols. 10. Policy papers, guidelines and legislation without any distinct connection to healthy ageing. 11. Psychometric or feasibility/pilot studies. 12. Papers based on individual opinions without reference to literature or research. 13. Abstract is missing; no access to abstract and/or full text. 14. Full text is missing. 15. Conference papers. 16. Resumés or comments on article or book (second opinion).

**ID = intellectual disability**

### Databases and search strategy

The information specialist and librarian from Lund University (MB) conducted a systematic search in 11 databases. No time limit was set for the publication of studies and all studies indexed in the selected databases up to 1 January 2024 were included. The search was conducted on 2022–04–15, then updated 2024–02–14 in the following databases: PubMed, Cinahl Complete, APA PsycInfo, SocIndex, Urban Studies Abstracts, ERIC, Academic Search Complete, Scopus, Cochrane Library, Web of Science Core Collection and Embase (Supplementary Material 2).

### Study selection

**Fig. 1:**
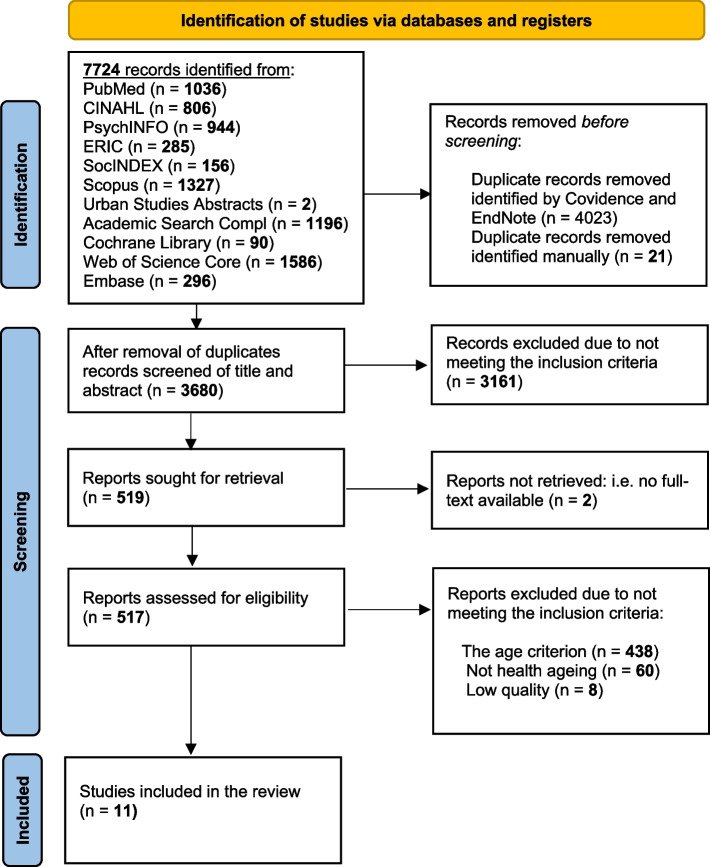
Search process according to the PRISMA 2020

The identified studies (*n* = 5010**)** were imported into the web-based collaboration software platform Covidence [32] by the information specialist (MB). Duplicates of studies were removed via this platform and later by the researchers in the review process (*n* = 1333). Two of the authors independently screened the articles in each step and excluded those that did not meet the eligibility criteria (Fig. 1). Disagreements in the assessment of studies were resolved through consultation with the third author — or, in the case of conflict of interest, through consultation with an external researcher outside the project but with knowledge of reseach on ID. In parallel to the screening process there were regular meetings between the three authors on the interpretation of the PICO protocol, either face-to-face or via Zoom (Zoom Video Communications, Inc.). In order to minimise bias in the review process, written working guidelines on how to apply the PICO principles and exclusion criteria were developed at these meetings.

### Quality assessment

The full-text articles included had different research designs. It was therefore decided to use the CASP (Critical Appraisal Skills Programme, 2024) checklists developed for different designs [33]. The quality assessment was carried out in two steps. In the first step, a CASP checklist was selected for the current study design and its items were checked off with complementary comments about the quality. In the second step, the completed checklist was used to determine a quality level for each full-text article: low (to be excluded), moderate or high (Table 2). These quality levels were developed within the research team as they are not included in the CASP guidelines. Two of the authors independently assessed quality, a third author was consulted in the case of disagreement on quality and the external researcher was involved in the case of conflict of interest. At this stage, the studies on policies, guidelines and legislation were found to be of low quality and therefore were excluded.

**Table 2:** Criteria for the assessment of quality level developed for the particular study after appraisal through the CASP

**Low or very low quality (to be excluded)**
1. The purpose and/or the research questions are not clearly described.
2. The method chosen is not suited to the purpose/questions.
3. The method is not clearly described.
4. The sampling of persons is not clearly described.
5. The criteria for inclusion are either not described or not suited to the purpose/questions.
6. Application for ethical approval is not mentioned despite the vulnerability of the group being sampled.
7. In the description of the results no distinction is made between different groups within the sample.
8. The results are not in line with the purpose/questions.
9. The results are not in line with the study design.
10. In the discussion of the method there is no account of the method’s weaknesses.
11. There is no description of the data underlying the recommendations for healthy ageing.
**Medium quality (to be included if all the requirements are met)**
1. The purpose and the research questions are clearly described.
2. The method chosen is suited to the purpose/questions.
3. The method is clearly described.
4. The criteria for inclusion are suited to the purpose/questions.
5. Ethical approval has been granted.
6. The results are in line with the purpose/questions.
7. The results are in line with the study design.
8. There is an account of the method’s weaknesses.
9. There is a partial description of the data underlying the recommendations for healthy ageing.
**High quality (which means that the following requirements must be fulfilled in addition to those designated for medium quality)**
1. Background, Method, Results, Discussion and Conclusions are well-described, coherent and relevant to healthy ageing.
2. The discussion brings out clearly the present state of empirical and theoretical knowledge concerning healthy ageing and similar concepts.
3. The discussion brings out clearly the existent gaps in such knowledge.
4. There is a clear description of the methodological limitations concerning validity, reliability and generalisability (or, in the case of qualitative studies, credibility, dependability, confirmability and transferability).
5. There is a description of the implications concerning future research, practice and education.

### Data extraction and data analysis

Data extraction was performed independently by two of the authors, using the research questions as the extraction template. A textual narrative synthesis was chosen to integrate the findings from both qualitative and quantitative studies. The method is appropriate when the aim is to describe the scope of what has been studied, the strength of the evidence available and the gaps that need to be filled [28, 34, 35]. Step 1 of the analysis involved making an overview of each study that corresponded to the inclusion criteria according to PICO (Table 1). Step 2 involved extracting the text concerning the healthy-ageing perspective and the text of the answer for each research question in each study. Step 3 involved condensing the text in order to further clarify the focus on results that offer a high or moderate degree of confidence.

## Results

### Overview of included studies

After the removal of duplicates, 3,677 titles and abstracts were screened, whereby 3,161 studies were excluded as they did not meet the inclusion criteria (Figure 1). The remaining 516 studies were reviewed in full-text, whereby 505 were excluded for reasons such as not meeting the age criterion or having their focus on ageing but not on *healthy* ageing, not clearly defining the terms used or simply being of a generally low quality. Thus there remained 11 studies which met our inclusion criteria, two were of high quality and the other 9 were of moderate quality.

Out of the 11 studies (Table 3), 9 were qualitative in design, using mainly individual interviews and focus group discussions. These studies explored aspects of healthy ageing from the perspectives of both individuals with ID, care providers and managers. One study [36] employed a mixed-methods approach that included surveys and interviews with individuals with and without ID, care staff and major stakeholders across the disability and aged-care sectors. One of the studies was a systematic review [37], which categorised 23 articles as concerning interventions for healthy ageing. These articles did not use the same definitions/terms as in this study but the systematic review did so.

**Table 3:** Overview of the studies (assessed as middle or high quality according to CASP checklists) concerning healthy ageing

**Authors, year of publication, title, journal name, volume (no), pages and doi-link**	**Aim**	**Country**	**Study design**	**Participants (number, age, sex)**	**Intervention** **(content, period, intensity etc.)**	**Comparison/** **control group (number of participants, age, sex etc.)**	**Outcome measures; healthy ageing and synonymous terms**	**Main results**
[42]. Ambivalence among staff regarding ageing with intellectual disabilities: Experiences and reflections *Journal of Intellectual Disabilities* 2021: 25(2); 192–209 10.1177/1744629519874997	To explore how staff in ID services experience and reflect on ageing in people with ID	Sweden	Qualitative and explorative	24 staff members, mean age 48 years (range: 24–65 years); 83% women, 17% men; average working experience in ID services 16.6 years (range: 2–41 years)			Ageing in place ^b^Healthy ageing, ^b^Successful ageing, ^b^Ageing well	Staff expressed ambivalence regarding ageing with ID, there were uncertainty, contradictions and inconsistency. They found it difficult to separate the ageing process from the disability itself. The ageing process in people with ID seemed to be the same as in people without ID
[44]. What it’s like to grow older: the aging perceptions of people with an intellectual disability in Ireland *Intellectual and Developmental Disabilities*: 52(3); 205–219. 10.1352/1934-9556-52.3.205	To explore and describe the perceptions of aging among people with ID, including their concerns and expectations, and to present a comprehensive representation of their aging experience	Ireland	Sub-study of the national longitudinal study IDS-TILDA Face-to-face interviews based on 7 closed and 5 open questions	367 people with ID and/or their proxies Mean age 54.1 years, range 41–90 years. 57.4% women, 43.6% men			Good things about aging ^b^Aging well is a part of active aging and successful aging ^b^Healthy aging	The opportunity to actively engage in life, both physically and socially, was important to the participants. Retirement, wisdom, fulfilment and independence represent what they believe life will be like as they age
[40]. Service providers’ perceptions of active ageing among older adults with lifelong intellectual disabilities. *Journal of Intellectual Research*: 56; 1133–1147. 10.1111/j.1365-2788.2011.01500.x	Two main aims: 1) to identify service providers’ experiences, views and perceptions of ageing and achieving active ageing among older adults with lifelong ID and 2) to better understand what active ageing might entail, both now and in the future, and the implications for older people, families, caregivers and service providers	Australia	Qualitative research design; semi-structured interviews	16 direct-care staff in two Australian states, Queensland and Victoria. They had worked with the older adult with ID who had nominated them for interview Most were female but it is not stated how many, nor are their ages given			Active ageing analyzed based on WHOs six core for active ageing (2000) ^b^Successful ageing	Service providers found them to play a primary role in promoting active ageing. The older adults need to make choices between alternative and desired activities, maintaining a feeling of purpose and involvement in social interactions and in the community. Facilitating was important when older adults had a strong desire to stay active and learn new things
[41]. Ageing in place together: older parents and ageing offspring with intellectual disability. *Ageing & Society*: 42; 480–494. 10.1017/S0144686X20001038	To explore which older parents chose ageing in place together with their ageing offspring with ID instead of other options and what factors are associated with this choice	Taiwan	A survey through structured face-to-face interviews. Regression analysis included variables of the four domains of Clapham’s [49] housing pathways framework	237 parents from older two-generation families, 76% mothers. The mean age of the parents was 75.4 years (range 60–99) and that of the cohabiting offspring with ID was 48.6 (range 40–74)		Comparison of the group of those ageing in place (*n* = 146, 61.6%) with the group who chose another option (*n* = 91, 38.4%)	Ageing in place	About 62% of the parents chose ageing in place, while 38% chose another option. Ageing in place was associated with personal control (housing ownership), increased self-identity (satisfaction with the living community), and self-esteem (life- satisfaction)
[45]. Good life in old age: Qualitative interviews about ageing with older adults with mild intellectual disability, prior to an educational intervention. *Journal of Intellectual Disabilities*: 10.1177/17446295231213689	To investigate how people with ID experience ageing, prior to an educational intervention	Sweden	Exploratory qualitative design based on qualitative interviews	The total number of participants was 26; 17 (65.4%) female, aged 42‒74 years (mean 61.3)			Meaningful ageing ^b^Healthy ageing	The main findings gave rise to two themes. The first “Live for today, tomorrow you are old” speaks for the avoidance of ageing, for living in the present and for working as long as possible. The second, “Need of support to enable a meaningful ageing”, speaks for the future. Meaningful ageing included activities after retirement, with a need of support from the staff
[36]. Mitigating the impact of the ‘silos’ between the disability and aged-care sectors in Australia: Development of a Best Practice Framework. *Journal of applied Research:* 34:1477–1488. 10.1111/jar.12890	To proposes a Best Practice Framework to mitigate the silos of the aged-care and disability service sectors	Australia	Mixed-method; Survey and interviews	Survey: People with ID (*n* = 391), mean age 65.2; 37.3% women Interviews: Policymakers and senior managers (*n* = 35) Interviews: People with and without ID (*n* = 39), age range 50–79 years, 60% women		People without ID (*n* = 920), mean age 71.9, 67.9% women	Successful Community-based Ageing ^b^Successful ageing, ^b^Ageing-in-my-Chosen-Place, ^b^Ageing-in-place, ^b^Quality of life	The participants indicated the importance of developing integrated care systems. Key components are choice as to accommodation; regular assessment of health and well-being indicators; development of policies across integrated aged- and disability-care sectors; improved strategies for workforce planning; and upskilling of existing staff including collaboration
[38]. Healthy Ageing in People with Intellectual Disability from Managers’ Perspective: A Qualitative Study *Healthcare* 5; 45. 10.3390/healthcare5030045	To explore healthy ageing among people with ID from the leaders’ perspective in ID services	Sweden	Qualitative and explorative	20 leaders, 4 of them middle-line leaders and 16 front-line leaders. Fifteen were leaders for group homes and five for daily activity centres. Five were men and fifteen women			Healthy ageing ^b^Ageing well	Healthy ageing for older with ID implied living according to their preferences and making independent choices. Leisure activities needed to be accessible daytime for those who no longer went to a daily activity centre. Ageing was described in terms of physical and cognitive changes. The need of support was highly individual, and the ageing was same for men and women
[43]*. *The key elements of ageing well: Perspectives of middle-aged adults with intellectual disabilities and family carers in South Korea. Journal of intellectual & developmental disability 47(3): 265–275. 10.3109/13668250.2021.1985333	To better understand the key elements of and the barriers to ageing well, based on the perspectives of both middle-aged adults with ID and their families	South Korea	Qualitative, in-depth interviews	10 middle-aged adults with ID and 12 family carers. The middle-aged adults with ID were 46–61 years old, mean age 54.7 years; 8 male and 2 females. Family caregivers were 45‒84 years old, mean age 68.5 years; 3 males and 9 females. Seven parents and five siblings participated as family carers			Ageing well ^b^Healthy ageing, ^b^Active ageing	The key elements of ageing well were living in a familiar place, having a reliable carer, nurturing independence, staying fit and healthy, and actively engaging in social activities The overarching desire in both groups was to have more choice in the preparation for their later lives and adequate resources
[46]. Age-friendly communities for older persons with intellectual disabilities. *Quality in Ageing and older adults:* 20(4);206–208. 10.1108/QAOA-11-2018-0058	To understand how an age-friendly Canadian city, Winnipeg, Manitoba is from the perspectives of older people with ID and their caregivers Furthermore, to increase awareness of the needs of older people with ID by making recommendations to support healthy and active ageing of this vulnerable population	Canada	Qualitative interviews on eight age-friendly domains [50]. Consisted of 7 individual interviews with older people with ID and 3 focus groups with family caregivers, employed service providers and support workers	Seven persons with mild ID, aged 45–57 years, six women and one man. Also 15 family carers and paid employees participated			Age-friendly community, based on eight domains [50]: transportation, housing, social participation, respect and social inclusion, community involvement, communication and information, community support and healthcare services, and outdoor space and buildings ^b^Healthy ageing, ^b^Active ageing, ^b^Ageing in place	The results reveal that the structure of the city’s physical environment (e.g. transportation system and housing options) contributed to confident mobility and helped to foster independent living and support ageing in place. The results indicated that many of the current features of the city of Winnipeg do not adequately address the needs of ageing people with ID; specifically, issues related to accessibility, social participation, social disrespect and inclusion, and lack of resources were important barriers to independence
[37]. Efficacy of Healthy Aging Interventions for Adults with Intellectual and Developmental Disabilities: A Systematic Review *The Gerontologist*: 62(4); 235–252 10.1093/geront/gnaa192	To provide an overview of healthy aging strategies/interventions for adults with IDD and to determine to what degree these strategies/ interventions are effective	USA (*n* = 10) ^a^, Australia (*n* = 2) ^a^, Taiwan (*n* = 2) ^a^, Canada (*n* = 1) ^a^, Israel (*n* = 1) ^a^; Europe: UK (*n* = 1) ^a^, Scotland (*n* = 3)^ a^, Italy (*n* = 1) ^a^, Netherlands (*n* = 1) ^a^, Northern Ireland (*n* = 1) ^a^	Systematic literature review carried out in the databases Web of Science, Scielo and PsycINFO with no limitation of time period	Examined 23 studies: 15 quantitative nonrandomized, 5 randomized and 3 qualitative. Concerned totally 2,398 adults with ID (range 8–379 participants) of both sexes, aged 18–86 years (mean 44.3 years)	Areas of interventions: (a) physical activity and health nutrition (*n* = 10), (b) health education and health screening (*n* = 6), (c) social inclusion and community participation (*n* = 3), and (d) multicomponent (*n* = 4)	Four randomized controlled studies had control group. Three of these studies with people with ID [51, 56, 57,55]. One the study included also people without ID [56]	Healthy ageing [22, 25] ^b^Health promotion	It was not possible to more precisely determine the effectiveness of the interventions because of the different designs and measures. However, interventions that address multiple domains including education, preventive screening programmes and knowledge about healthy lifestyle are most likely to be effective. Follow-up studies were lacking and the healthy aging interventions were scarce, incipient and sporadic
[39]. What is and isn’t working: Factors involved in sustaining community‐based health and participation initiatives for people ageing with intellectual and developmental disabilities. *Journal of Applied Research in Intellectual Disabilities* (JARID): 32;1465–1477. https://doi.org/10.1111/jar.12640	To explore supports and barriers in respect of sustaining community‐ based health and participation initiatives (CBHPIs) for people ageing with IDD living in group homes managed by agencies	Chicago, USA	Qualitative design utilising semi‐structured interviews with managers and staff and photovoice method, i.e. discussions based on photographs taken of people with IDD	Of 35 professionals 6 were managers and 29 staff; mean age 43, range 18–65; 76% women, 24% men, and 35 participants with IDD; mean age 52, range 26–98, 53% women and 47% men		Similarities in experiences across the two participating groups are highlighted in the results	Healthy ageing ^b^Health promotion	Agency values and policies related to healthy ageing; resources and staff competencies; communication between management and staff; community/ University partnerships; and peer relations. Despite value of CBHPIs, they are difficult to sustain due to limited resources and lack of training specific to ageing with IDD

^a^Numbers within parenthesis in the column Country for Santos et al. 2022 are the numbers of studies in the different countries

*ID* Intellectual disability, *IDD *Intellectual and developmental disability

^b^The term is used synonymously with the main term in the study

The total number of participants in the 11 studies was 3,938, presented here in three different groups. The great majority (3,234, 82%), were older adults with ID, with a minimum of 7 to a maximum of 2,398 in the particular study. The number of care staff working in ID services (i.e. direct care staff, leaders and policymakers), was 455 (12%) in total, with a minimum of 15 and a maximum of 310 in the particular study. The number of informal caregivers (family members) was 249 (6%) in total, with a minimum of 12 and a maximum of 237 in the particular study (see Table 3). The age range of the participants with ID was 18‒98 years, whilst that of the care staff and informal caregivers was 24‒99 years (Table 3).

### Concepts and definitions of healthy ageing

The term “healthy ageing” was mainly highlighted in three [37–39] of the eleven studies, referring to the WHO definition of healthy ageing [22]. Healthy ageing was for Santos and colleagues [37] a question of maintaining functional ability and well-being into older age. Furthermore this systematic review was able to identify healthy-ageing interventions, and their effectiveness was evaluated in terms of integrating healthy-living concepts, reducing health-related problems and improving the lifestyle of people with ID. In the study conducted by Johansson and colleagues [38], the leaders perceived healthy ageing in terms of enabling people to live in accordance with their preferences and make independent choices, supporting leisure activities and providing support to meet increased needs due to ageing. Spassiani and colleagues [39] focused on the agency values and policies related to healthy ageing, such as supporting health promotion, developing formal policies and supporting community-based health and participation initiatives. Thus the concern with healthy ageing in Spassiani and colleagues’ study [39] is mainly on the organisational and societal level.

### Concepts and definitions similar to healthy ageing found in the studies

Most authors used several terms in their study, often as being synonymous with healthy ageing (Table 3). The distinction between the terms used was not clarified in every study. The main terms used were the following: active ageing [40], ageing in place [41, 42], ageing well [43], good things about ageing [44], meaningful ageing [45], age-friendly communities [46] and successful community-based ageing [36]. The other terms used are shown in Table 3, and when these were treated as synonyms they are marked.

#### Active ageing

Active ageing was highlighted by Buys and colleagues [40] in the form of community interaction, personal and social engagement, involving for instance the development of life-enhancing skills and the pursuit of health-promoting activities. The study focused on identifying the care staff’s experience of active ageing among older adults with ID. The outcomes were analysed in accordance with the six categories described by the WHO [47]: (1) *Development of Practical, Leisure or Life-enhancing Skills*: care staff described active ageing among people with ID as being able to make choices between alternative activities and access community opportunities for work or retirement. (2) *Improved or Maintained Dietary and General Health Status*: care staff saw age-related problems as being viewed as a natural part of the ageing process, though such problems did have a negative impact on the general health of the older adults with ID. (3) *Varied Rhythm of Life*: care staff described the importance of participation in desired activities as critical for the quality of life of older adults with ID. (4) *Recognition that Challenge and Productivity Must Continue Throughout Old Age*: care staff described the importance of maintaining a feeling of purpose and involvement through old age for older adults with ID. (5) *Increased and Well-established Social Network*: care staff described the importance of regular social interaction with friends and acquaintances. (6) *Regular Participation in the General Life of the Community*: regular planned activities in external settings; external organisations facilitate participation in giving older adults with ID the opportunity to engage in activities accompanied by the care staff.

#### Ageing in place

Ageing in place was explored by Chou and Kröger [41] on the basis of the concept of housing pathways [48, 49]. The meaning of home for people involves experiences of security, positive identity (both self-identity and social identity) and self-esteem, which contribute to the ability to cope and a sense of ownership. In Chou and Kröger’s study [41] more than half of the older parents chose to age in place with their ageing offspring with ID. This was associated with personal control (housing ownership), increased self-identity (satisfaction with the living community) and self-esteem (life-satisfaction).

Ageing in place was described by Alftberg and colleagues [42] as enhancing life and well-being for older adults with ID through the continuity, stability and comfort provided by a familiar environment. This environment enhances the possibility for older adults with ID to maintain their independence and autonomy. Another factor when it comes to ageing in place is the setting of the group home, which includes residents of different ages. On the positive side, the younger residents can encourage older residents to stay active in their daily life. However, the negative side is that older residents describe the presence of younger residents in the group home as exhausting.

#### Ageing well

Kim and colleagues [43] considered ageing well in terms of the importance of preparation for later life. They identified five themes concerning ageing well: living in a familiar place, having a reliable carer, nurturing independence, staying fit and healthy, and actively engaging in social activities. *Living in a familiar place* developed a sense of safety and comfort and maintained a close proximity with family carers. *Having a reliable carer* ensured a continuous sense of security and facilitated preparation for later life. *Nurturing independence* paved the way to independent living. *Staying fit and healthy* facilitated the avoidance of age-related health problems. Finally, *actively engaging in social activities* maintained social participation and community involvement.

#### Good things about ageing

The term “good things about ageing”, employed by Burke and colleagues [44], covered themes such as social activities, wisdom, fulfilment, independence and retirement. *Social activities* such as travelling, dancing, and walking were highly appreciated. *Wisdom* was appreciated in terms of both offering and gaining knowledge. *Fulfilment* and *independence* allowed people to enjoy doing tasks around the house and made them feel more confident and more able to make their own decisions*. Retirement* was seen as positive in that it meant more relaxation and free time. These themes together contributed to the positive experience of healthy ageing on the part of people with ID.

#### Meaningful ageing

One study highlighted the concept of meaningful ageing. Holmgren and Ahlström [45], defined it as the continuation of leisure activities and maintaining working as long as possible. They identified four key aspects of meaningful ageing: *Grasping the transition to becoming old*, which meant that the older adults with ID saw ageing as bringing greater wisdom but also as being associated with particular health problems and with dementia. The aspect *Difficulty in anticipating retirement* had to do with such things as how to spend one’s days and worries about loneliness. *Desire for enjoyable activities in older age* included looking forward to participating in enjoyable activities despite the difficulty of taking the initiative oneself. When it comes to *Expectation of being able to decide accommodation*, the participants expressed the need to live in a supportive and social community and have access to the accommodation that suited their needs as they grew older.

#### Age-friendly communities

The WHO term “age-friendly communities” was used by Miskimmin and colleagues [46] and was based on the following eight age-friendly organisational and societal domains [50]: transportation, housing, social participation, respect and social inclusion, communication and information, community support and healthcare services, civic participation and employment, and outdoor space and buildings. Each domain was described in terms of opportunities and barriers.

*Transportation* can offer opportunities in terms of convenience, affordability and independence; and can offer barriers in terms of fixed schedule, accessibility issues during winter, long waiting and travel times. *Housing* can offer opportunities in terms of design (modern and with fewer stairs, for instance) and location (being close to essential services, for instance); and can offer barriers in the same areas. *Social participation* can offer opportunities in terms of availability and the support of carers; and can offer barriers in terms of lack of transportation, exclusion (such as not being asked to join clubs) and lack of variety of social activities. When it comes to *respect and social inclusion,* participants generally felt respected and accepted. However, decreased mobility could be a barrier and increase their social isolation. When it comes to *community and information*, being kept up to date about community events is an important component of active ageing. Barriers are complete reliance on staff for receiving information and poor access to internet and technology. *Community support and healthcare services* can offer obvious opportunities but there can be barriers such as lack of information on how to age successfully, difficulty in communicating with staff and changes in the services available. *Civic participation and employment* can offer opportunities leading to an increased feeling of involvement but there can be barriers in the form of, for instance, limited options for volunteer work or paid employment, or difficulty being paid. *Outdoor space and buildings* can offer opportunities in terms of, for instance, accessibility (such as lighter doors, push buttons to open doors). Barriers can be such things as poor snow removal, slippery pavements and limited rest areas.

#### Successful community-based ageing

The term “successful community-based ageing” focuses on the organisational and societal levels. It was highlighted by Hussain and colleagues [36] as an outcome, focusing on integrated services that reduce the separate sectors (“silos”) between the disability and aged-care sectors for older adults with and without ID. The four themes of successful community-based ageing were choice and autonomy, health and well-being, service improvement and flexible accommodation options*. Choice and autonomy* allowed individuals to make decisions about their living arrangements. *Health and well-being* were supported through measurable indicators used in regular assessments and targeted services. *Service improvement* was promoted through national policies and standards to improve the service access and targeted services for people with complex needs. *Flexible accommodation options* provided people with the ability to live in their preferred settings. These themes contribute to better quality of life and outcomes for older adults, both with and without ID.

### Healthy-ageing research concerning older adults with intellectual disability at the individual, organisational and societal levels

Outcomes of healthy-ageing interventions at the individual level were found in the systematic review performed by Santos and colleagues [37]. The 23 studies included were described in terms of four themes based on the main content of the interventions: *Physical activity and health nutrition, Health education and health screening, Social inclusion and community participation* and *Multicomponent interventions*. Twenty of the studies had a quantitative design (15 nonrandomised and 5 randomised controlled trials), the other three had a qualitative design. Five interventions included follow-up measures, and these were undertaken between 3 and 12 months after [51–55]. Of the 23 studies only four, or 17%, had a between-subject design with control group [51, 55–57], the other 19, or 83%, had a within-subject design.

Ten of the 23 studies concerned *Physical activity and health nutrition*, with main focus on the frequency of physical activity of older adults with ID. The average duration of the interventions was 18.5 weeks (range 5‒36 weeks). The intervention consisted of one to four sessions per week. All studies showed significant results, except in the case of two interventions, the “Walk Well Intervention” and the “Healthy Eating Video — Supported Program”. The main outcomes included increased physical activity and improved fitness as well as weight loss.

Furthermore, six intervention studies of *Health education and health screening* addressed health education with regard to physical activity and fitness, nutrition and diet, health screening knowledge and self-sufficiency. The most common duration was 6–8 weeks, though one intervention lasted 12 months. The number of sessions varied between 1 and 8 per week (except in the case of the long intervention, which had 4 sessions). Some interventions significantly increased health knowledge, including screening knowledge, improved self-management and reduced health risks such as that of diabetes.

Moreover, three of the 23 intervention studies focused on *Social inclusion and community participation*. These interventions had a long duration, ranging from 6 to 29 months. The sessions varied between 4 h a week and once a month. Two of the three interventions showed a significant increase in participation in community groups, new social contacts and increased social satisfaction among the participants.

Finally, the four *Multicomponent interventions* studies using a mixed approach with several components lasted 12 weeks and one 7 months (Healthy Lifestyle Change Program). There were two or three sessions per week. All four showed significant psychological and attitudinal outcomes, with weight loss and improved nutritional habits, increased physical activity and greater life-satisfaction with a somewhat lower depression rate.

Although the effect sizes of the interventions were calculated, the authors [37] concluded that it was not possible to decide definitively which intervention was the most efficacious because of the different aims, designs and measured outcomes. However, the findings support the notion that education and preventive screening programmes as well as knowledge about healthy lifestyle are potentially the most successful healthy-ageing resources for individuals with ID. In addition, the study concluded that interventions that address multiple domains are more likely to be effective in promoting healthy ageing than are interventions that focus on a single domain [37].

Regarding studies on the organisational and societal levels for older adults with ID, we included two that were qualitative and one that was mixed-method. Spassiani and colleagues [39] explored supports and barriers in respect of sustaining community-based health and participation initiatives (CBHPIs) for people ageing with ID living in group homes managed by agencies. Miskimmin and colleagues [46] explored features of, and facilitators and barriers concerning, age-friendly communities from the perspectives of older adults with mild ID and family carers. The study by Hussain and colleagues [36] proposed a “Best Practice Framework” with the goal of better supporting community-based successful ageing of older adults with and without ID.

### Healthy ageing in developing and developed countries and continents

All healthy-ageing studies were conducted in developed countries. This meant that it was not possible to answer the research question posed in this study about similarities and differences in the description of healthy ageing between developing and developed countries and continents. Three studies included were conducted in Sweden [38, 42, 45], two in Australia [36, 40] and one in each of the following countries: USA [39], Canada [46], Taiwan [41], South Korea [43] and Ireland [44]. Furthermore the systematic review carried out by Santos and colleagues [37] included studies from eight developed countries but none from developing countries. The developed countries were the USA, the UK, Australia, Taiwan, Canada, Israel, the Netherlands and Italy.

### Participatory research and the public involvement of older adults with intellectual disability

The large majority of the research planning, design and analysis was done by the researchers without involvement of the older adults with ID. In the case of participatory research, the study carried out by Burke and colleagues [44] the older adults with ID played an active role in the design by participating in focus groups and pilot testing, which supported the development of research questions that were appropriate and meaningful for them. Furthermore, in the Holmgren and Ahlström study [45] the involvement of the participants in the development of an educational intervention in respect of ageing ensured that the content of the educational programme was in line with the needs of older adults with ID. Spassiani and colleagues [39] used photovoice, allowing older adults with ID to capture images representing their experiences. These photographs were then used in the data collection to facilitate group discussions and the analysis. Moreover, most of the articles, namely Buys and colleagues [40], Chou and colleagues [41], Kim and colleagues [43], Santos and colleagues [37] and Miskimmin and colleagues [46], focus on user, family and professional engagement to collect data and present findings.

A low degree of public involvement was presented by Hussain and colleagues [36]. This covered stakeholders as policymakers, senior managers, healthcare professionals and individuals with ID. Burke and colleagues [44] showed collaboration between researchers and an advocacy group of people with ID and their families who helped to review and prepare the study materials. Spassiani and colleagues [39] emphasized public involvement by engaging nonprofit agencies to identify group homes that had ageing residents with ID, also they provided input to ensure that the methods were appropriate for the participants. To sum up, the participants’ involvement did not include participation in the designing, conducting or analyzing of the research.

### Ethical considerations and issues in connection with healthy-ageing research

The focus of ethical considerations in the included studies concerned general research issues in respect of people with ID, not specifically healthy ageing. The most common concern was informed consent to participate in the studies, which was mentioned in the method section in relation to ethical approval but was not highlighted further in the discussion section.

Holmgren and Ahlström [45] utilised methods of informed consent, pre-recorded video, easy-reading written information and repeated oral information to ensure that the participants understood what it meant to participate in the study. The importance of informed consent is emphasised by Burke and colleagues [44], who focused on the inclusion of people with all levels of ID. The plain and simple language of the illustrated information booklet on the study and of the informed-consent form contributed to ensuring that participation was voluntary.

Hussain and colleagues [36] included only participants who understood English and could speak for themselves and give informed consent, i.e. persons with mild to moderate ID. The authors highlight the dilemma that people with severe/profound ID are mostly excluded from research due to their cognitive and communication limitations. This exclusion also affects people from cultural and linguistical backgrounds different from those of the country where the study is taking place. The authors discuss the concern as to whether the language restriction in their study also contributes to this marginalisation and isolation of older people with severe/profound ID, and in particular of those from cultural and linguistically diverse backgrounds. Spassiani and colleagues [39] also included only older adults with mild to moderate ID, and they used legal guardians to provide written consent for those who did not have the capacity to provide it themselves. Miskimmin and colleagues [46] gave persons who were eligible for their study the interview questions in advance, together with an information letter about the study to ensure informed consent.

### Knowledge gaps within healthy-ageing research and interventions for older adults with intellectual disability

The studies included in our systematic scoping review concerning healthy ageing were few in number (*n* = 11) and had different aims, designs and results. The fact that the qualitative studies and the survey study constituted 10 out of the 11 studies means that there is a knowledge gap when it comes to randomised control trials. Therefore the results cannot be combined in a meta-analysis to draw conclusions, which means that there is a knowledge gap concerning the efficacy of the healthy-ageing interventions. Another clear knowledge gap that emerged had to do with the fact that few authors elaborated their theoretical perspective on healthy ageing in their background and rationale for the study design.

Santos and colleagues [37] concluded in their systematic review of studies of healthy-ageing interventions for people with ID that these studies came only from developed countries. The age criteria for inclusion were within the range 18–86 years but very few studies focused on participants aged 60 or older. The designs of the 23 studies were mainly nonrandomised (22% [*n* = 5] were randomised controlled trials) and the sample sizes were mainly small. Most of the interventions showed a temporary positive effect. The same intervention was not replicated in different samples; and follow-ups were uncommon, which meant knowledge gaps concerning the long-term effects. Furthermore, most of the interventions concerned only people with mild and moderate ID, therefore little is known about the effect of an intervention on people with severe ID. Also, few interventions were carried out on the basis of the interests and the needs of people with ID; most of the interventions were designed by researchers without involvement of these people as co-researchers in the research process [37]. Our systematic scoping review and that of Santos and colleagues [37] come to the same conclusion: more preliminary studies with a clearer perspective on healthy ageing are needed.

## Discussion

The results of this scoping review show that few empirical studies concerning older adults with ID apply a theoretical perspective in respect of healthy ageing. It was found that several terms could be used in the same study to signify healthy ageing, in some cases with a clear explanation of the nuances represented by the different terms but most often not. Our review had no geographical limitations but only studies from developed countries met the inclusion criteria, limiting the generalisability of the findings. The studies differed with regard to design (it was mostly qualitative) and group of participants, and they can therefore be considered exploratory and hypothesis-generating. Furthermore, our review confirms Santos and colleagues’ conclusion in their systematic review of studies of healthy-ageing interventions for people with ID that such studies are scarce, incipient and sporadic [37].

The results of this scoping review identified gaps in knowledge concerning healthy ageing, which were also highlighted as a rationale for the development of the WHO Action Plan for the Decade of Healthy Ageing 2021–2030 [25]. The aim of this action plan is that by 2030 great progress shall have been made in the use of evidence-based approaches and the optimising older people’s functional ability, which is the key to healthy ageing. The WHO has defined functional ability in the following terms: “1) ability to meet one’s basic needs; 2) ability to learn, grow and make decisions; 3) mobility; 4) ability to build and maintain relationships; and 5) ability to contribute. Functional ability combines the intrinsic capacity of the individual, the environment a person lives in and how people interact with their environment” [25], page Xiii).

The WHO proposes optimising older people’s functioning through four key actions: 1) changing the way we think, feel and act about age and ageing; 2) ensuring that communities support older people’s abilities; 3) providing person-centred, integrated care and primary health services that meet older people’s needs; and 4) access to long-term care for older people who need it [25]. Critical are the capacity for integrated action across sectors, connecting stakeholders so that they can share experiences and learn from each other, and strengthening data, research and innovation to foster healthy ageing. There is also a need for more comparable data on healthy ageing, for more evaluation of programmes and for new technologies that can help to meet the needs and expectations of older people [25]. Our results highlight the need to develop evidence-based interventions to optimise the functional capacity for those with ID, in line with the 2030 deadline.

To achieve evidence-based interventions, new studies are needed, with research across countries and disciplines involving older people [25]. However, the researcher must take account of the cognitive impairment of the person with ID before asking them whether they would be willing to participate in research. A recent systematic review highlighted the importance of informed consent prior to participation in research [58]. When it comes to the participation of persons with ID it is critical that researchers use an approach informed by a proper awareness of disability rights in order to enhance consent capacity and ensure responsible inclusion. However, there is only limited evidence on best practice for informed consent, and the available literature focuses only on approaches to improving understanding and providing decision support. Challenges and failures in the informed consent process, as well as an overemphasis on protection, often contribute to the exclusion of adults with ID. For this population with health inequalities, these barriers needed to be overcome [58]. Two studies in our review considered the complexity of obtaining informed consent from these people [44, 45]. This complexity needs to be better described in future studies, as do the ways researchers go about obtaining informed consent from people with ID.

The shift towards involving people with ID as research partners is commonly described in the literature by means of such terms as participatory research, participatory action research, inclusive research, co-design or co-production [59]. When there is such involvement, the results are usually more relevant and meaningful as these people contribute first-hand insights that can reveal aspects of their needs and daily lives that might otherwise be overlooked [59, 60]. Several reviews published in recent years have identified both the benefits and challenges of inclusive or participatory research [59, 61, 62]. One of the challenges is that additional resources are needed to develop relation-based collaboration and to ensure understanding of the research, as well as to address issues of communication and accessibility for people with ID [59, 60]. To help with this, researchers can for example develop simplified questions and visual aids, or involve supportive persons who can assist with understanding [44, 45]. In one of the studies in our review the older adults with ID were involved in developing the content of an intervention that will be evaluated in a future study [45]. However, the review revealed that there is still room for improvement when it comes to inclusive research practices in respect of healthy ageing in line with the WHO guidelines [25] for people with ID.

The studies in this review present clear methods used for participatory research such as focus groups and photovoice but are less informative on methods for public involvement. Public involvement in research means that the research is done by or in conjunction with the public, which in turn means that the patients or other people with relevant experience contribute to how the research is designed, conducted and disseminated [63]. For example, frameworks such as INVOLVE (UK) provide helpful guidance for embedding public involvement in research practices, whereby stakeholders and people with lived experience have an important role to play the research process [64, 65]. The studies we encountered presented only limited public involvement and there is therefore a need to increase the contribution of relevant groups of people from different sectors of society as active collaborators in healthy-ageing research, this in order to enhance the relevance and quality of such research.

All the results in our review originated from developed countries. That we in this same field know far less about developing countries may be understood in terms of the barriers which exist in these countries. Research from developing countries reveals that cultural and religious beliefs have a great impact on the lives of people with ID in terms of stigma and discrimination, curses, punishment and social exclusion [66–69]. This puts a burden on families and it limits access to healthcare and support networks; there is a barrier in the form of other people’s fear, shame and judgement [66, 67, 69].

Despite increasing awareness of the human rights of people with ID [27] the lack of policy implementation, resources and trained professionals has been described as an ongoing problem in developing countries [70, 71]. Economic barriers such as poverty in conjunction with a high cost of living make it difficult to access basic needs such as food, transportation and healthcare [66, 72–74]. Treatment is left to untrained practitioners, who are unable to meet the complex needs of people with ID [70, 71]. Ensuring health equity for persons with disabilities has been laid down as a global priority for policymakers and research [26]. Addressing the issues involved in the implementation of measures to support healthy ageing among people with ID, especially in societies with limited resources, is thus an important challenge for researchers.

### Methodological considerations

This scoping review has some limitations that need to be taken into account when interpreting the results. To begin with, only studies written in English were included. Furthermore most of the studies in the review process did not meet the inclusion criteria in respect of the definition of healthy ageing, the perspective on it or the age of the participants. Our results are limited to older adults and cannot be generalised to younger ones. However, the large number of studies excluded due to the limitation of the healthy-ageing perspective and the age criterion contributed to our decision to make an exception and include the systematic review by Santos and colleagues [37]. This review made a categorisation of healthy-ageing interventions and had a mean age close to the inclusion criterion, i.e. 44.3 years. That study was the only one we found that gave information about interventions from the perspective of healthy ageing. Santos and colleagues [37] results confirmed our finding that there are few healthy-ageing interventions for older adults with ID. It is important to be aware, though, that the results from that review are not valid when it comes to people with severe or profound ID, as the interventions mainly involved only people with mild to moderate ID [37].

In addition, a relatively large number of epidemiological studies, as also a number of studies with other designs, were excluded because they did not have a clear healthy-ageing perspective. Our decision to take account not just of the term “healthy ageing” but also of closely related terms enhances the finding that there exists a large knowledge gap today in respect of the implementation of the perspective of healthy ageing for this target group. Therefore it needs to be taken into consideration that this scoping review does not include empirical studies without a defined healthy-ageing perspective or ones based solely on diagnoses and disease conditions. This is an important limitation to the generalisability of the results. However, we hope that future studies will define and explain their perspective on healthy ageing, as health is a multidimensional concept which is used in many ways [22–24]. It is important to be aware of the health perspective of the population with intellectual disability, who live with physical, cognitive and social disabilities from early life. The identified studies in this review mainly used qualitative methods, and the quantitative interventions were quite heterogeneous in terms of research questions and outcome measures. A further factor which should be considered when interpreting the results is that the interventions included in the review conducted by Santos and colleagues [37] varied in intensity and duration. This heterogeneous picture of relatively few interventions significantly reduces the possibility of drawing conclusions about the validity and generalisability of the identified healthy-ageing interventions.

The difference in study design was a hindrance to performing a meta-analysis, which is a further limitation of this study. Critical appraisal to discover risk of bias was performed in the case of each study design by at least two researchers using the CASP guidelines [33]. However, such a risk arose in that two of the authors contributed to studies that met the inclusion criteria [75, 76]. This threat to good research practice was addressed by using an external researcher who received all written information about the purpose of the project, the research questions and the inclusion and exclusion criteria. This external researcher was asked to remain impartial in conducting an independent review. The authors themselves were aware of the risk of bias and took a critical approach to the findings.

Our way to be transparent regarding the weaknesses is reporting by the PRISMA-ScR Checklist 2020 items, which facilitates replication and systematic review [29, 30]. Summarising the characteristics of studies to a synthesis and describing the uncertainty of the evidence in the results helps researchers and research-funding organisations to formulate appropriate recommendations for future research practice [29].

## Conclusions

This scoping review portrays the landscape of healthy-ageing research for the vulnerable group of older adults with ID. It confirms that healthy-ageing interventions are few in number, sporadic and predominantly non-randomised, with varying research questions and variable intensity and duration of the interventions. Whether the research is qualitative or quantitative, it highlights the challenges posed by different definitions of healthy ageing, which threaten comparability and generalisability. The methodological limitations and knowledge gaps identified should encourage future researchers to prioritise the healthy-ageing perspective in research, improve the evidence-based interventions and increase the involvement of older adults with ID as partners in research.

## Supplementary information

Supplementary Material 1.

Supplementary Material 2.

## Data Availability

The literature were stored in Covidence platform.

## Abbreviations

ID: Intellectual disability
PICO protocol: Patient/population, Intervention, Comparison/control, and Outcome
PRISMA: Preferred Reporting Items for Systematic reviews and Meta-Analyses
PRISMA-ScR: PRISMA guidelines for Scoping Reviews

## References

[1] Haveman M, Salvador-Carulla L J, Walsh PN, Kerr M, Lantman-de Valk Schrojenstein H, et al. Ageing and health status in adults with intellectual disabilities: Results of the European POMONA II study. Journal of Intellectual & Developmental Disability. 2011;36(1):49–60. 21314593 10.3109/13668250.2010.549464

[2] Martínez-Leal R, Salvador-Carulla L, Linehan C, Walsh P, Weber G, Van Hove G, et al. The impact of living arrangements and deinstitutionalisation in the health status of persons with intellectual disability in Europe. J Intell Disabil Res. 2011;55(9):858–72. 10.1111/j.1365-2788.2011.01439.x PMC3166640 21726319

[3] O’Leary L, Cooper S-A, Hughes-McCormack L. Early death and causes of death of people with intellectual disabilities: A systematic review. J Appl Res Intellect. 2018;31(3):325–42. 10.1111/jar.12417 28984406

[4] Reppermund S, Srasuebkul P, Dean K, Trollor JN. Factors associated with death in people with intellectual disability. J Appl Res Intellect. 2020;33(3):420–9. 10.1111/jar.12684 31786826

[5] Axmon A, Ahlström G, Westergren H. Pain and pain medication among older people with intellectual disabilities in comparison with the general population. Healthcare. 2018;6(2):67. 29914061 10.3390/healthcare6020067 PMC6023323

[6] De Leeuw MJ, Oppewal A, Elbers RG, Knulst MW, Van Maurik MC, Van Bruggen MC, et al. Healthy Ageing and Intellectual Disability study: summary of findings and the protocol for the 10-year follow-up study. BMJ Open. 2022;12:e053499. 10.1136/bmjopen-2021-053499. 35193910 10.1136/bmjopen-2021-053499 PMC8867312

[7] Hansford R, Ouellette-Kuntz H, Bourque MA, Decker K, Derksen S, Hallet J, et al. Investigating inequalities in cancer staging and survival for adults with intellectual or developmental disabilities and cancer: A population-based study in Manitoba. Canada Cancer Epidemiology. 2024;88: 102500. 38035452 10.1016/j.canep.2023.102500

[8] Mazza MG, Rossetti A, Crespi G, Clerici M. Prevalence of co-occurring psychiatric disorders in adults and adolescents with intellectual disability: A systematic review and meta-analysis. J Appl Res Intellect. 2020;33(2):126–38. 10.1111/jar.12654 31430018

[9] McCarron M, Cleary E, McCallion P. Health and health-care utilization of the older population of Ireland: comparing the intellectual disability population and the general population. Res Aging. 2017;39(6):693–718. 28566009 10.1177/0164027516684172

[10] Sandberg M, Ahlström G, Kristensson J. Patterns of somatic diagnoses in older people with intellectual disability: a Swedish eleven year case–control study of inpatient data. J Appl Res Intellect. 2017;30(1):157–71. 10.1111/jar.12230 26542759

[11] Axmon A, Björne P, Nylander L, Ahlström G. Psychiatric diagnoses in older people with intellectual disability in comparison with the general population: a register study. Epidemiology and psychiatric sciences. 2018;27(5):479–91. 28228177 10.1017/S2045796017000051 PMC6137377

[12] Coppus AM. People with intellectual disability: what do we know about adulthood and life expectancy? Dev Disabil Res Rev. 2013;18(1):6–16. 23949824 10.1002/ddrr.1123

[13] Dieckmann F, Giovis C, Offergeld J. The life expectancy of people with intellectual disabilities in Germany. J Appl Res Intellect. 2015;28(5):373–82. 10.1111/jar.12193 26256274

[14] Ng N, Flygare Wallén E, Ahlström G. Mortality patterns and risk among older men and women with intellectual disability: a Swedish national retrospective cohort study. BMC Geriatr. 2017;17(1):269. 29166873 10.1186/s12877-017-0665-3 PMC5700486

[15] Glover G, Williams R, Heslop P, Oyinlola J, Grey J. Mortality in people with intellectual disabilities in England. J Intell Disabil Res. 2017;61(1):62–74. 10.1111/jir.12314 27489075

[16] Robertson J, Heslop P, Lauer E, Taggart L, Hatton C. Gender and the Premature Deaths of People with Intellectual Disabilities: An International Expert Consultation. Journal of Policy and Practice in Intellectual Disabilities. 2021;18(2):89–103.

[17] Torr J, Strydom A, Patti P, Jokinen N. Aging in Down Syndrome: Morbidity and Mortality. Journal of Policy and Practice in Intellectual Disabilities. 2010;7(1):70–81.

[18] Bertelli MO, Fletcher R, Weber G, Schuengel C, Scuticchio D, Bianco A, et al. Psychological distress and physical vulnerability. Textbook of Psychiatry for Intellectual Disability and Autism Spectrum Disorder: Springer; 2022. p. 71–94.

[19] Bigby C. Known well by no-one: Trends in the informal social networks of middle-aged and older people with intellectual disability five years after moving to the community. J Intellect Dev Disabil. 2008;33(2):148–57. 18569402 10.1080/13668250802094141

[20] Carmeli E, Imam B. Health promotion and disease prevention strategies in older adults with intellectual and developmental disabilities. Front Public Health. 2014;2:31. 24783190 10.3389/fpubh.2014.00031 PMC3995041

[21] Heller T, Fisher D, Marks B, Hsieh K. Interventions to promote health: crossing networks of intellectual and developmental disabilities and aging. Disabil Health J. 2014;7(1):S24–32. 24456681 10.1016/j.dhjo.2013.06.001

[22] WHO. World report on ageing and health 2015 [Available from: https://iris.who.int/bitstream/handle/10665/186463/9789240694811_eng.pdf?sequence=1.

[23] Beard JR, Officer AM, Cassels AK. The world report on ageing and health. Gerontologist. 2016;56(S2):S163–6. 10.1093/geront/gnw037. 26994257 10.1093/geront/gnw037

[24] WHO. Global strategy and action plan on ageing and health. 2017 [Available from: https://www.who.int/publications/i/item/9789241513500.

[25] WHO. Decade of healthy ageing: baseline report. E. World Health Organization. 2020 [Available from: https://www.who.int/publications/i/item/9789240017900.

[26] WHO. Global report on health equity for persons with disabilities. E. World Health Organization 2022 [Available from: https://www.who.int/publications/i/item/9789240063600.

[27] United Nations. Convention on the Rights of Persons with Disabilities (CRPD). 2006 [Available from: https://www.un.org/development/desa/disabilities/convention-on-the-rights-of-persons-with-Disabilities.html. 10.1515/9783110208856.203 18348362

[28] Tricco AC, Antony J, Soobiah C, Kastner M, MacDonald H, Cogo E, et al. Knowledge synthesis methods for integrating qualitative and quantitative data: a scoping review reveals poor operationalization of the methodological steps. J Clin Epidemiol. 2016;73:29–35. 26891948 10.1016/j.jclinepi.2015.12.011

[29] Page MJ, McKenzie JE, Bossuyt PM, Boutron I, Hoffmann TC, Mulrow CD, The PRISMA, et al. statement: an updated guideline for reporting systematic reviews. BMJ (Clinical research ed). 2020;2021:372. 10.1136/bmj.n71 PMC8005924 33782057

[30] Tricco AC, Lillie E, Zarin W, O’Brien KK, Colquhoun H, Levac D, et al. PRISMA extension for scoping reviews (PRISMA-ScR): checklist and explanation. Ann Intern Med. 2018;169(7):467–73. 30178033 10.7326/M18-0850

[31] James Thomas DK, Joanne E McKenzie, Sue E Brennan, Soumyadeep Bhaumik. Chapter 2: Determining the scope of the review and the questions it will address. Cochrane 2023. 2023 [Available from: https://training.cochrane.org/handbook/current/chapter-02#section-2-3.

[32] Covidence. Covidence systematic review software, Veritas Health Innovation, Melbourne, Australia. 2024 [Available from: www.covidence.org.

[33] CASP. Critical Appraisal Skills Checklists. 2024 [Available from: https://casp-uk.net/casp-tools-checklists/.

[34] Essex R, Bruce G, Dibley M, Newton P, Thompson T, Swaine I, Dibley L. A systematic scoping review and textual narrative synthesis of the qualitative evidence related to adolescent idiopathic scoliosis. International Journal of Orthopaedic and Trauma Nursing. 2022;45: 100921. 35217471 10.1016/j.ijotn.2022.100921

[35] Lucas PJ, Baird J, Arai L, Law C, Roberts HM. Worked examples of alternative methods for the synthesis of qualitative and quantitative research in systematic reviews. BMC Med Res Methodol. 2007;7:1–7. 17224044 10.1186/1471-2288-7-4 PMC1783856

[36] Hussain R, Parmenter T, Wark S, Janicki M, Knox M, Hayhoe N. Mitigating the impact of the ‘silos’ between the disability and aged-care sectors in Australia: Development of a Best Practice Framework. J Appl Res Intellect. 2021;34(6):1477–88. 10.1111/jar.12890 34046986

[37] Santos FH, Zurek J, Janicki MP. Efficacy of Healthy Aging Interventions for Adults With Intellectual and Developmental Disabilities: A Systematic Review. Gerontologist. 2020;62(4):e235–52. 10.1093/geront/gnaa192 33220058

[38] Johansson M, Björne P, Runesson I, Ahlström G. Healthy ageing in people with intellectual disabilities from managers’ perspective: a qualitative study. Healthcare. 2017;5(3):45. 10.3390/healthcare5030045. 28820435 10.3390/healthcare5030045 PMC5618173

[39] Spassiani NA, Meisner BA, Abou Chacra MS, Heller T, Hammel J. What is and isn’t working: Factors involved in sustaining community-based health and participation initiatives for people ageing with intellectual and developmental disabilities. J Appl Res Intellect. 2019;32(6):1465–77. 10.1111/jar.12640 31264333

[40] Buys L, Aird R, Miller E. Service providers’ perceptions of active ageing among older adults with lifelong intellectual disabilities. J Intell Disabil Res. 2012;56(12):1133–47. 10.1111/j.1365-2788.2011.01500.x 22044681

[41] Chou Y-C, Kröger T. Ageing in place together: older parents and ageing offspring with intellectual disability. Ageing Soc. 2022;42(2):480–94.

[42] Alftberg Å, Johansson M, Ahlström G. Ambivalence among staff regarding ageing with intellectual disabilities: Experiences and reflections. J Intellect Disabil. 2021;25(2):192–209. 31570036 10.1177/1744629519874997 PMC8120636

[43] Kim HS, Lee CE, Kim KM. The key elements of ageing well: Perspectives of middle-aged adults with intellectual disabilities and family carers in South Korea. J Intellect Dev Disabil. 2022;47(3):265–75. 39818570 10.3109/13668250.2021.1985333

[44] Burke E, McCarron M, Carroll R, McGlinchey E, McCallion P. What it’s like to grow older: the aging perceptions of people with an intellectual disability in Ireland. Ment Retard. 2014;52(3):205–19. 10.1352/1934-9556-52.3.205 24937746

[45] Holmgren M, Ahlström G. Good life in old age: Qualitative interviews about ageing with older adults with mild intellectual disability, prior to an educational intervention. J Intellect Disabil. 2024;28(4):1118–36. 10.1177/17446295231213689. 37950579 10.1177/17446295231213689 PMC11585186

[46] Miskimmin C, Shooshtari S, Menec V, Duncan KA, Martin T, Stoesz BM. Age-friendly communities for older persons with intellectual disabilities. Quality in Ageing and Older Adults. 2019;20(4):206–18.

[47] WHO. Healthy ageing–adults with intellectual disabilities: summative report. J Appl Res Intellect. 2001;14(3):256–75.

[48] Clapham D. Housing pathways: A post modern analytical framework. Hous Theory Soc. 2002;19(2):57–68.

[49] Clapham D. Happiness, well-being and housing policy. Policy Polit. 2010;38(2):253–67.

[50] WHO. Global age-friendly cities: A guide. 2007 [Available from: https://iris.who.int/bitstream/handle/10665/43755/9789241547307_eng.pdf?sequence=1.

[51] Lennox N, Bain C, Rey-Conde T, Purdie D, Bush R, Pandeya N. Effects of a comprehensive health assessment programme for Australian adults with intellectual disability: a cluster randomized trial. Int J Epidemiol. 2007;36(1):139–46. 17218326 10.1093/ije/dyl254

[52] Aronow HU, Hahn JE. Stay well and healthy! pilot study findings from an inhome preventive healthcare programme for persons ageing with intellectual and/or developmental disabilities. J Appl Res Intellect. 2005;18(2):163–73.

[53] Lunsky Y, Straiko A, Armstrong S. Women be healthy: Evaluation of a women’s health curriculum for women with intellectual disabilities. J Appl Res Intellect. 2003;16(4):247–53.

[54] Melville CA, Boyle S, Miller S, Macmillan S, Penpraze V, Pert C, et al. An open study of the effectiveness of a multi-component weight-loss intervention for adults with intellectual disabilities and obesity. Br J Nutr. 2011;105(10):1553–62. 21255473 10.1017/S0007114510005362

[55] Taggart L, Truesdale M, Carey M, Martin-Stacey L, Scott J, Bunting B, et al. Pilot feasibility study examining a structured self-management diabetes education programme, DESMOND-ID, targeting HbA1c in adults with intellectual disabilities. Diabet Med. 2018;35(1):137–46. 29083501 10.1111/dme.13539

[56] Ewing G, McDermott S, Thomas-Koger M, Whitner W, Pierce K. Evaluation of a cardiovascular health program for participants with mental retardation and normal learners. Health Educ Behav. 2004;31(1):77–87. 14768659 10.1177/1090198103259162

[57] Matthews L, Mitchell F, Stalker K, McConnachie A, Murray H, Melling C, et al. Process evaluation of the Walk Well study: a cluster-randomised controlled trial of a community based walking programme for adults with intellectual disabilities. BMC Public Health. 2016;16:1–11. 27387203 10.1186/s12889-016-3179-6 PMC4936049

[58] McDonald KE, Schwartz AE, Dinerstein R, Olick R, Sabatello M. Responsible inclusion: a systematic review of consent to social-behavioral research with adults with intellectual disability in the US. Disabil Health J. 2024;17(4). 10.1016/j.dhjo.2024.101669. 10.1016/j.dhjo.2024.101669 PMC13285379 38960791

[59] Hewitt O, Langdon PE, Tapp K, Larkin M. A systematic review and narrative synthesis of inclusive health and social care research with people with intellectual disabilities: How are co-researchers involved and what are their experiences? J Appl Res Intellect. 2023;36(4):681–701. 10.1111/jar.13100 37002721

[60] Bigby C. Social inclusion and people with intellectual disability and challenging behaviour: A systematic review. J Intellect Dev Disabil. 2012;37(4):360–74. 23002899 10.3109/13668250.2012.721878

[61] Di Lorito C, Bosco A, Birt L, Hassiotis A. Co-research with adults with intellectual disability: A systematic review. J Appl Res Intellect. 2018;31(5):669–86. 10.1111/jar.12435 29231285

[62] Frankena TK, Naaldenberg J, Cardol M, Linehan C, van Schrojenstein Lantman-de Valk H. Active involvement of people with intellectual disabilities in health research–A structured literature review. Research in developmental disabilities. 2015;45:271–83. 26280692 10.1016/j.ridd.2015.08.004

[63] Health Research Authority (HRA). Public involvement in NHS 2025 [Available from: https://www.hra.nhs.uk/planning-and-improving-research/best-practice/public-involvement/

[64] National Institutes for Health and Care Research (NIHR). Briefing notes for researchers - public involvement in NHS, health and social care research 2021 [Available from: https://www.nihr.ac.uk/briefing-notes-researchers-public-involvement-nhs-health-and-social-care-research.

[65] National Institute for Health Research (NHS). Public involvement in systematic reviews: Supplement to the briefing notes for researchers. INVOLVE, Eastleigh. Report 2012. Available 14 Feb 2025: https://training.cochrane.org/sites/training.cochrane.org/files/public/uploads/resources/downloadable_resources/INVOLVE%202012%20PublicInvolvementSystematicReviews.pdf.

[66] Jansen-van Vuuren J, Aldersey H. Stigma, acceptance and belonging for people with IDD across cultures. Curr Dev Disord Rep. 2020;7:163–72. 32837827 10.1007/s40474-020-00206-w PMC7326393

[67] Opoku MP, Elhoweris H, Jiya AN, Ngoh NA-P, Nketsia W, Kumi EO, Torgbenu EL. Cross-national study of communal attitudes toward individuals with intellectual disabilities in sub-Saharan Africa: Cameroon vs. Ghana Plos one. 2021;16(9):e0257482. 34582489 10.1371/journal.pone.0257482 PMC8478177

[68] Disability stigma in developing countries. K4D Helpdesk Report. Brighton: Institute of Development Studies. Available from: 13 Feb 2025. https://assets.publishing.service.gov.uk/media/5b18fe3240f0b634aec30791/Disability_stigma_in_developing_countries.pdf.

[69] Sango PN. Country profile: Intellectual and developmental disability in Nigeria. Tizard Learning Disability Review. 2017;22(2):87–93.

[70] Memari AH, Hafizi S. People with intellectual disability and social–political life participation: A commitment to inclusive policies in less developed countries. Journal of Policy and Practice in Intellectual Disabilities. 2015;12(1):37–41.

[71] Parmenter TR. The present, past and future of the study of intellectual disability challenges in developing countries. Salud pública de méxico. 2008;50(2):124–31. 10.1590/s0036-36342008000800004 18470339

[72] Belachew A, Cherbuin N, Bagheri N, Burns R. A Systematic Review and Meta-analysis of the Socioeconomic, Lifestyle, and Environmental Factors Associated with Healthy Ageing in Low and Lower-Middle-Income Countries. J Popul Ageing. 2024;17(2):365–87. 10.1007/s12062-024-09444-x.

[73] Eleweke CJ, Ebenso J. Barriers to accessing services by people with disabilities in Nigeria: Insights from a qualitative study. J Educ Soc Res. 2016;6(2). 10.5901/jesr.2016.v6n2p113.

[74] Gréaux M, Moro MF, Kamenov K, Russell AM, Barrett D, Cieza A. Health equity for persons with disabilities: a global scoping review on barriers and interventions in healthcare services. International Journal for Equity in Health. 2023;22(1):236. 37957602 10.1186/s12939-023-02035-w PMC10644565

[75] Vetenskapsrådet. Good Research Practice. Swedish Research Council. 2024 [Available from: https://www.vr.se/english/mandates/ethics/ethics-in-research.html.

[76] All European Academies. The European Code of Conduct for Research Integrity 2023 [Available from: https://allea.org/code-of-conduct/.

