# Supplementary material for: Healthy ageing for older adult people with intellectual disability: a scoping review
Source: Arch Public Health. 2025 Feb 27;83:55. doi: 10.1186/s13690-025-01528-0 (PMC11866571; doi:10.1186/s13690-025-01528-0)
Supplement: Supplementary file 2 — Supplementary Material 2. [file 13690_2025_1528_MOESM2_ESM.pdf]

Matthias Bank, Librarian / Information specialist
Library & ICT, Faculty of Medicine
Lund University
Matthias.Bank@med.lu.se

LUND UNIVERSITY
Faculty of Medicine

SUPPLEMENTARY MATERIAL 2: SEARCH REPORT "ID AND HEALTHY AGING"

PROJECT INFORMATION

REQUEST DATE	NAME	INSTITUTION/ORGANISATION
2022-04-15	Gerd Ahlström, Nadia El Mrayyan	Department of Health Sciences, Lund University

Updated 2024-02-14 (see page 10 in this document)

DESCRIBE RESEARCH QUESTION AND PURPOSE WITH THE SEARCH

1. What is the definition of the term healthy ageing in the literature concerning older adult people with intellectual disability?
2. What similar concepts to healthy ageing are used in the literature?
3. How is healthy ageing promoted through research for older adults with intellectual disability on individual, organizational and societal level?
4. What are the similarities and differences in the description of healthy ageing between developing and developed countries and continents?
5. How is participatory research used in the healthy-ageing research for older adults with intellectual disability?
6. Are ethical issues or dilemmas taken up in connection with healthy ageing?
7. What are the knowledge gaps within healthy ageing research and interventions for older adult people with intellectual disability?

STRUCTURED RESEARCH QUESTION

PROVIDE THE RESEARCH QUESTION IN A STURCTURED FORMAT :PICO(S), PEO OR IN FREE ORDER	
Patient / population / problem	Adults 45 and over with ID
Intervention	
Comparison / control	
Outcome	Healthy aging
Studytype(s)	

SEARCH STRATEGIES

The search strategy documentation is to be cited as below, for protocol or publication in journal as appendix. The documentation is structured according to PRISMA 2020, international standard for systematic reviews. Please note that the information specialist delivers the parts required for search strategy documentation, additional method information may also be required.

See PRISMA 2020 for further information:
http://prisma-statement.org/
http://prisma-statement.org/Extensions/Searching

Databases searched:

Academic Search Complete, APA PsycInfo, Cinahl Complete, Cochrane Library, Embase, ERIC, PubMed, Scopus, SocIndex, Urban Studies Abstracts, Web of Science Core Collection

---

PubMed (National Library of Medicine)
Date of search: 2022-05-27

#1 ("Intellectual Disability"[Mesh] OR "intellectual disabilit*" OR "Down Syndrome"[Mesh] OR "Down Syndrome" OR "intellectual abnormalit*" OR "learning abnormalit*" OR "mental abnormalit*" OR “neurodevelopmental abnormali*” OR "intellectual deficienc*" OR "learning deficienc*" OR "mental deficienc*" OR “neurodevelopmental deficienc*” OR "mental retardation" OR "developmental disabilit*" OR "learning disabilit*") 172,986

#2 (aged[MeSH] OR Aged[TI] OR adult*[TI] OR older[TI] OR "middle aged"[Mesh] OR “middle aged”[TW] OR ageing[TW] OR aging[TW] OR elderly[TW] OR “old age”[TW] OR elder[TW] OR senior*[TW] OR “older adult*”[TW] OR “over 45” [TW] OR "over 50"[TW] OR "over 55“[TW] OR "over 60"[TW] OR "over 65"[TW] OR “over 70” [TW] OR “over 75” [TW] OR "over 80"[TW] OR “over 85” [TW] OR "45 and over"[TW] OR "50 and over"[TW] OR "55 and over"[TW] OR "60 and over"[TW] OR "65 and over"[TW] OR "70 and over"[TW] OR "75 and over"[TW] OR "80 and over"[TW] OR "85 and over"[TW] OR geriatric[TW] OR quinquagenarian*[TW] OR sexagenarian*[TW] OR septuagenarian*[TW] OR octogenarian*[TW] OR nonagenarian*[TW]) 6,167,340

#3 ("Healthy Aging"[Mesh] OR "healthy aging" OR "healthy ageing" OR "ageing well" OR "aging well" OR "well ageing" OR "well aging" OR “ageing in place” OR “aging in place” OR "successful ageing" OR "successful aging" OR "healthy living" OR "health indicator*" OR “health promotion” OR healthy[TI] OR policy[TI] OR policies[TI] OR prevention*[TI] OR intervention*[TI] OR guideline*[TI] OR legislation*[TI] OR act[TI] OR model[TI] OR law[TI] OR laws[TI] OR promotion[TI] OR promoting[TI]) 1,282,208

#4 #1 AND #2 AND #3 1,267

#5 #4 NOT (child[TI] OR children[TI] OR infant*[TI] OR “young adult*”[TI] OR mouse[TI] OR mice[TI] OR murine[TI] OR genetic[TI] OR childhood[TI] OR tissue*[TI] OR rat[TI] OR rats[TI] OR prenatal[TI] OR antenatal[TI]) 975

#6 #5 NOT (animals[MH] NOT humans[MH]) 953
Filters: English 921
The search filter for older/elderly people was adjusted from the Canadian Health Libraries Association, https://extranet.santecom.qc.ca/wiki/!biblio3s/doku.php?id=concepts:personnes_agees

CINAHLComplete (Cumulative Index to Nursing and Allied Health Literature; EbscoHost, inception to present)

Date of search: 2022-05-30

S1	((MH "Intellectual Disability+") OR (MH "Down Syndrome")) OR ("intellectual disabilit*" OR "Down Syndrome" OR "intellectual abnormalit*" OR "learning abnormalit*" OR "mental abnormalit*") OR (“neurodevelopmental abnormali*” OR "intellectual deficienc*" OR "learning deficienc*" OR "mental deficienc*" OR “neurodevelopmental deficienc*” OR "mental retardation" OR "developmental disabilit*" OR "learning disabilit*")	58,269
S2	((MH "Aged+") OR (MH "Middle Age")) OR TI (aged OR adult* OR older) OR ("middle aged" OR ageing OR aging OR elderly OR "old age" OR elder OR senior* OR "older adult*" OR "over 45" OR "over 50" OR "over 55") OR ("over 60" OR "over 65" OR "over 70" OR "over 75" OR "over 80" OR "over 85" OR "45 and over" OR "50 and over" OR "55 and over" OR "60 and over" OR "65 and over" OR "70 and over" OR "75 and over" OR "80 and over" OR "85 and over") OR (geriatric OR quinquagenarian* OR sexagenarian* OR septuagenarian* OR octogenarian* OR nonagenarian*)	1,639,381
S3	((MH "Healthy Aging") OR "healthy aging" OR "healthy ageing" OR "ageing well" OR "aging well" OR "well ageing" OR "well aging" OR “ageing in place” OR “aging in place”) OR ("successful ageing" OR "successful aging" OR "healthy living" OR "health indicator*" OR “health promotion”) OR TI (healthy OR policy OR policies OR prevention* OR guideline* OR legislation* OR act OR model OR law OR laws OR promotion OR promoting OR intervention*)	536,139
S4	S1 AND S2 AND S3	829
S5	(S1 AND S2 AND S3) NOT TI (child OR children OR infant* OR "young adult*" OR mouse OR mice OR murine OR genetic OR childhood OR tissue* OR rat OR rats OR prenatal OR antenatal)	728
S6	Filter: English	725

APA PsycInfo (EbscoHost, inception to present)

Date of search: 2022-05-30

S1	((DE "Learning Disabilities") OR (DE "Down's Syndrome")) OR ("intellectual disabilit*" OR "Down Syndrome" OR "intellectual abnormalit*" OR "learning abnormalit*" OR "mental abnormalit*") OR (“neurodevelopmental abnormali*” OR "intellectual deficienc*" OR "learning deficienc*" OR "mental deficienc*" OR “neurodevelopmental deficienc*” OR "mental retardation" OR "developmental disabilit*" OR "learning disabilit*")	112,742
S2	((DE "Older Adulthood") OR (DE "Middle Adulthood")) OR TI (aged OR adult* OR older) OR ("middle aged" OR ageing OR aging OR elderly OR "old age" OR elder OR senior* OR "older adult*"	639,227

	OR "over 45" OR "over 50" OR "over 55") OR ("over 60" OR "over 65" OR "over 70" OR "over 75" OR "over 80" OR "over 85" OR "45 and over" OR "50 and over" OR "55 and over" OR "60 and over" OR "65 and over" OR "70 and over" OR "75 and over" OR "80 and over" OR "85 and over") OR (geriatric OR quinquagenarian* OR sexagenarian* OR septuagenarian* OR octogenarian* OR nonagenarian*)	
S3	((DE "Healthy Aging") OR (DE "Aging in Place") OR "healthy aging" OR "healthy ageing" OR "ageing well" OR "aging well" OR "well ageing" OR "well aging" OR “ageing in place” OR “aging in place”) OR ("successful ageing" OR "successful aging" OR "healthy living" OR "health indicator*" OR “health promotion”) OR TI (healthy OR policy OR policies OR prevention* OR guideline* OR legislation* OR act OR model OR law OR laws OR promotion OR promoting OR intervention*)	368,190
S4	S1 AND S2 AND S3	1,110
S5	(S1 AND S2 AND S3) NOT TI (child OR children OR infant* OR "young adult*" OR mouse OR mice OR murine OR genetic OR childhood OR tissue* OR rat OR rats OR prenatal OR antenatal)	899
S6	Filter: English	885

SocIndex (EbscoHost, inception to present)
Date of search: 2022-05-30

S1	((DE "Learning Disabilities") OR (DE "MENTAL disabilities")) OR ("intellectual disabilit*" OR "Down Syndrome" OR "intellectual abnormalit*" OR "learning abnormalit*" OR "mental abnormalit*") OR (“neurodevelopmental abnormali*” OR "intellectual deficienc*" OR "learning deficienc*" OR "mental deficienc*" OR “neurodevelopmental deficienc*” OR "mental retardation" OR "developmental disabilit*" OR "learning disabilit*")	15,600
S2	(DE "OLDER people") OR (DE "MIDDLE-aged persons" OR DE "MIDDLE age" OR DE "OLD age") OR TI (aged OR adult* OR older) OR ("middle aged" OR ageing OR aging OR elderly OR "old age" OR elder OR senior* OR "older adult*" OR "over 45" OR "over 50" OR "over 55") OR ("over 60" OR "over 65" OR "over 70" OR "over 75" OR "over 80" OR "over 85" OR "45 and over" OR "50 and over" OR "55 and over" OR "60 and over" OR "65 and over" OR "70 and over" OR "75 and over" OR "80 and over" OR "85 and over") OR (geriatric OR quinquagenarian* OR sexagenarian* OR septuagenarian* OR octogenarian* OR nonagenarian*)	150,732
S3	(DE "ACTIVE aging" OR DE "SUCCESSFUL aging") OR ("healthy aging" OR "healthy ageing" OR "ageing well" OR "aging well" OR "well ageing" OR "well aging" OR “ageing in place” OR “aging in place”) OR ("successful ageing" OR "successful aging" OR "healthy living" OR "health indicator*" OR “health promotion”) OR TI (healthy OR policy OR policies OR prevention* OR guideline* OR legislation* OR act OR model OR law OR laws OR promotion OR promoting OR intervention*)	202,880
S4	S1 AND S2 AND S3	157
S5	(S1 AND S2 AND S3) NOT TI (child OR children OR infant* OR "young adult*" OR mouse OR mice OR murine OR genetic OR childhood OR tissue* OR rat OR rats OR prenatal OR antenatal)	144
S6	Filter: English	144

Urban Studies Abstracts (EbscoHost, inception to present)
Date of search: 2022-05-30

#	Query	Results
S1	("intellectual disabilit*" OR "Down Syndrome" OR "intellectual abnormalit*" OR "learning abnormalit*" OR "mental abnormalit*") OR (“neurodevelopmental abnormali*” OR "intellectual deficienc*" OR "learning deficienc*" OR "mental deficienc*" OR “neurodevelopmental deficienc*” OR "mental retardation" OR "developmental disabilit*" OR "learning disabilit*")	127
S2	TI (aged OR adult* OR older) OR ("middle aged" OR ageing OR aging OR elderly OR "old age" OR elder OR senior* OR "older adult*" OR "over 45" OR "over 50" OR "over 55") OR ("over 60" OR "over 65" OR "over 70" OR "over 75" OR "over 80" OR "over 85" OR "45 and over" OR "50 and over" OR "55 and over" OR "60 and over" OR "65 and over" OR "70 and over" OR "75 and over" OR "80 and over" OR "85 and over") OR (geriatric OR quinquagenarian* OR sexagenarian* OR septuagenarian* OR octogenarian* OR nonagenarian*)	3,967
S3	(("healthy aging" OR "healthy ageing" OR "ageing well" OR "aging well" OR "well ageing" OR "well aging" OR “ageing in place” OR “aging in place”) OR ("successful ageing" OR "successful aging" OR "healthy living" OR "health indicator*" OR “health promotion”) OR TI (healthy OR policy OR policies OR prevention* OR guideline* OR legislation* OR act OR model OR law OR laws OR promotion OR promoting OR intervention*)	13,017
S4	S1 AND S2 AND S3	2
S5	(S1 AND S2 AND S3) NOT TI (child OR children OR infant* OR "young adult*" OR mouse OR mice OR murine OR genetic OR childhood OR tissue* OR rat OR rats OR prenatal OR antenatal)	2

ERIC (EbscoHost, inception to present)
Date of search: 2022-05-30

S1	(DE "Intellectual Disability" OR DE "Down Syndrome" OR DE "Mild Intellectual Disability" OR DE "Moderate Intellectual Disability" OR DE "Severe Intellectual Disability") OR ("intellectual disabilit*" OR "Down Syndrome" OR "intellectual abnormalit*" OR "learning abnormalit*" OR "mental abnormalit*") OR (“neurodevelopmental abnormali*” OR "intellectual deficienc*" OR "learning deficienc*" OR "mental deficienc*" OR “neurodevelopmental deficienc*” OR "mental retardation" OR "developmental disabilit*" OR "learning disabilit*")	54,233
S2	(DE "Older Adults”) OR TI (aged OR adult* OR older) OR ("middle aged" OR ageing OR aging OR elderly OR "old age" OR elder OR senior* OR "older adult*" OR "over 45" OR "over 50" OR "over 55") OR ("over 60" OR "over 65" OR "over 70" OR "over 75" OR "over 80" OR "over 85" OR "45 and over" OR "50 and over" OR "55 and over" OR "60 and over" OR "65 and over" OR "70 and over" OR "75 and over" OR "80 and over" OR "85 and over") OR (geriatric OR quinquagenarian* OR sexagenarian* OR septuagenarian* OR octogenarian* OR nonagenarian*)	85,703

S3	("healthy aging" OR "healthy ageing" OR "ageing well" OR "aging well" OR "well ageing" OR "well aging" OR “ageing in place” OR “aging in place”) OR ("successful ageing" OR "successful aging" OR "healthy living" OR "health indicator*" OR “health promotion”) OR TI (healthy OR policy OR policies OR prevention* OR guideline* OR legislation* OR act OR model OR law OR laws OR promotion OR promoting OR intervention*)	130,463
S4	S1 AND S2 AND S3	326
S5	(S1 AND S2 AND S3) NOT TI (child OR children OR infant* OR "young adult*" OR mouse OR mice OR murine OR genetic OR childhood OR tissue* OR rat OR rats OR prenatal OR antenatal)	273
S6	Filter: English	265

Academic Search Complete (EbscoHost, inception to present)

Date of search: 2022-05-27

S1	(DE "MENTAL disabilities") OR (DE "DOWN syndrome") OR ("intellectual disabilit*" OR "Down Syndrome" OR "intellectual abnormalit*" OR "learning abnormalit*" OR "mental abnormalit*") OR (“neurodevelopmental abnormali*” OR "intellectual deficienc*" OR "learning deficienc*" OR "mental deficienc*" OR “neurodevelopmental deficienc*”) OR ("mental retardation" OR "developmental disabilit*" OR "learning disabilit*")	115,472
S2	(DE "OLDER people" OR DE "MIDDLE age" OR DE "MIDDLE-aged persons" OR DE "OLD age") OR (TI (aged OR adult* OR older)) OR ("middle aged" OR ageing OR aging OR elderly OR "old age" OR elder OR senior* OR "older adult*" OR "over 45" OR "over 50" OR "over 55")) OR ("over 60" OR "over 65" OR "over 70" OR "over 75" OR "over 80" OR "over 85" OR "45 and over" OR "50 and over" OR "55 and over" OR "60 and over" OR "65 and over" OR "70 and over" OR "75 and over" OR "80 and over" OR "85 and over") OR (geriatric OR quinquagenarian* OR sexagenarian* OR septuagenarian* OR octogenarian* OR nonagenarian*)	1,231,767
S3	DE "ACTIVE aging" OR ("healthy aging" OR "healthy ageing" OR "ageing well" OR "aging well" OR "well ageing" OR "well aging" OR “ageing in place” OR “aging in place”) OR ("successful ageing" OR "successful aging" OR "healthy living" OR "health indicator*" OR “health promotion”) OR TI (healthy OR policy OR policies OR prevention* OR guideline* OR legislation* OR act OR model OR law OR laws OR promotion OR promoting OR intervention*)	1,555,056
S4	S1 AND S2 AND S3	1,356
S5	(S1 AND S2 AND S3) NOT (child OR children OR infant* OR "young adult*" OR mouse OR mice OR murine OR genetic OR childhood OR tissue* OR rat OR rats OR prenatal OR antenatal)	1,105
S6	Filter: English	1,093

Scopus

Date of search: 2022-05-27

#1 TITLE-ABS-KEY("intellectual disabilit*" OR "down syndrome" OR "intellectual abnormalit*" OR "learning abnormalit*" OR "mental abnormalit*" OR "neurodevelopmental abnormali*" OR "intellectual deficienc*" OR "learning deficienc*" OR "mental deficienc*" OR "neurodevelopmental deficienc*" OR "mental retardation" OR "developmental disabilit*" OR "learning disabilit*") 200,496

#2 (TITLE(aged OR adult* OR older) OR TITLE-ABS-KEY("middle aged" OR ageing OR aging OR elderly OR "old age" OR elder OR senior* OR "older adult*" OR "over 45" OR "over 50" OR "over 55" OR "over 60" OR "over 65" OR "over 70" OR "over 75" OR "over 80" OR "over 85") OR TITLE-ABS-KEY("45 and over" OR "50 and over" OR "55 and over" OR "60 and over" OR "65 and over" OR "70 and over" OR "75 and over" OR "80 and over" OR "85 and over") OR TITLE-ABS-KEY(geriatric OR quinquagenarian* OR sexagenarian* OR septuagenarian* OR octogenarian* OR nonagenarian*)) 5,719,245

#3 (TITLE-ABS-KEY("healthy aging" OR "healthy ageing" OR "ageing well" OR "aging well" OR "well ageing" OR "well aging" OR "ageing in place" OR "aging in place") OR TITLE-ABS-KEY("successful ageing" OR "successful aging" OR "healthy living" OR "health indicator*" OR "health promotion") OR TITLE(healthy OR policy OR policies OR prevention* OR intervention* OR guideline* OR legislation* OR act OR model OR law OR laws OR promotion OR promoting)) 3,871,264

#4 #1 AND #2 AND #3 1,594

#5 TITLE(child OR children OR infant* OR "young adult*" OR mouse OR mice OR murine OR genetic OR childhood OR tissue* OR rat OR rats OR prenatal OR antenatal) 3,897,290

#6 #4 AND NOT #5 1,229

#7 Filters: English 1,179

Cochrane Library via Cochrane Library Online (Wiley, Issue 5 of 12, May 2022)

Date of search: 2022-05-31

#1 MeSH descriptor: [Intellectual Disability] explode all trees 1,562

#2 MeSH descriptor: [Down Syndrome] explode all trees 405

#3 ("intellectual disabilit*" OR "Down Syndrome" OR "intellectual abnormalit*" OR "learning abnormalit*" OR "mental abnormalit*" OR “neurodevelopmental abnormali*” OR "intellectual deficienc*" OR "learning deficienc*" OR "mental deficienc*" OR “neurodevelopmental deficienc*” OR "mental retardation" OR "developmental disabilit*" OR "learning disabilit*"):ti,ab,kw (Word variations have been searched) 2,114

#4 #1 OR #2 OR #3 3,025

#5 MeSH descriptor: [Aged] explode all trees 220,635

#6 MeSH descriptor: [Middle Aged] explode all trees 333,649

#7 (Aged OR adult* OR older):ti (Word variations have been searched) 99,659

#8 ("middle aged" OR ageing OR aging OR elderly OR "old age" OR elder OR senior* OR "older adult*" OR "over 45" OR "over 50" OR "over 55"):ti,ab,kw OR ("over 60" OR "over 65" OR "over 70" OR "over 75" OR "over 80" OR "over 85" OR "45 and over" OR "50 and over" OR "55 and over" OR "60 and over"):ti,ab,kw OR ("65 and over" OR "70 and over" OR "75 and over" OR "80 and over" OR "85 and over"):ti,ab,kw OR (geriatric OR quinquagenarian*

OR sexagenarian* OR septuagenarian* OR octogenarian* OR nonagenarian*):ti,ab,kw (Word variations have been searched) 795,994

#9 #5 OR #6 OR #7 OR #8 817,471

#10 MeSH descriptor: [Healthy Aging] explode all trees 71

#11 ("healthy aging" OR "healthy ageing" OR "ageing well" OR "aging well" OR "well ageing" OR "well aging" OR “ageing in place” OR “aging in place” OR "successful ageing" OR "successful aging" OR "healthy living" OR "health indicator*" OR “health promotion”):ti,ab,kw (Word variations have been searched) 13,654

#12 (healthy OR policy OR policies OR prevention* OR guideline* OR legislation* OR act OR model OR law OR laws OR promotion OR promoting OR intervention*):ti (Word variations have been searched) 177,050

#13 #10 OR #11 OR #12 183,464

#14 #4 AND #9 AND #13 182

#15 (child OR children OR infant* OR "young adult*" OR mouse OR mice OR murine OR genetic OR childhood OR tissue* OR rat OR rats OR prenatal OR antenatal):ti,ab,kw (Word variations have been searched) 412,228

#16 #14 NOT #15 67

Web of Science Core collection (Clarivate Analytics)

Date of search: 2022-05-31

Databases searched in Web of Science Core Collection (inception as described below)

Science Citation Index Expanded (SCI-EXPANDED) --1900-present

Social Sciences Citation Index (SSCI) --1956-present

Arts & Humanities Citation Index (A&HCI) --1975-present

#1 "intellectual disabilit*" OR "down syndrome" OR "intellectual abnormalit*" OR "learning abnormalit*" OR "mental abnormalit*" OR "neurodevelopmental abnormali*" OR "intellectual deficienc*" OR "learning deficienc*" OR "mental deficienc*" OR "neurodevelopmental deficienc*" OR "mental retardation" OR "developmental disabilit*" OR "learning disabilit*" (Topic) 108,654

#2 aged OR adult* OR older (Title) or "middle aged" OR ageing OR aging OR elderly OR "old age" OR elder OR senior* OR "older adult*" OR "over 45" OR "over 50" OR "over 55" OR "over 60" OR "over 65" OR "over 70" OR "over 75" OR "over 80" OR "over 85" (Topic) or "45 and over" OR "50 and over" OR "55 and over" OR "60 and over" OR "65 and over" OR "70 and over" OR "75 and over" OR "80 and over" OR "85 and over" (Topic) or geriatric OR quinquagenarian* OR sexagenarian* OR septuagenarian* OR octogenarian* OR nonagenarian* (Topic) 4,926,540

#3 "healthy aging" OR "healthy ageing" OR "ageing well" OR "aging well" OR "well ageing" OR "well aging" OR "ageing in place" OR "aging in place" (Topic) or "successful ageing" OR "successful aging" OR "healthy living" OR "health indicator*" OR "health promotion" (Topic) or healthy OR policy OR policies OR prevention* OR intervention* OR guideline* OR legislation* OR act OR model OR law OR laws OR promotion OR promoting (Title) 3,545,553

#4 #1 AND #2 AND #3 2,232

#5 #4 NOT TI=(child OR children OR infant* OR "young adult*" OR mouse OR mice OR murine OR genetic OR childhood OR tissue* OR rat OR rats OR prenatal OR antenatal) 1,409

#6 Limit English 1,391

Embase.com (Elsevier, 1947-present)

Date of search: 2022-06-01

#01. 'mental deficiency'/exp OR 'down syndrome'/exp 158,862

#02. 'intellectual disabilit*':ti,ab OR 'down syndrome':ti,ab OR 'intellectual abnormalit*':ti,ab OR 'learning abnormalit*':ti,ab OR 'mental abnormalit*':ti,ab OR 'neurodevelopmental abnormalit*':ti,ab OR 'intellectual deficienc*':ti,ab OR 'learning deficienc*':ti,ab OR 'mental deficienc*':ti,ab OR 'neurodevelopmental deficienc*':ti,ab OR 'mental retardation':ti,ab OR 'developmental disabilit*':ti,ab OR 'learning disabilit*':ti,ab 104,895

#03. #1 OR #2 211,121

#04. 'aged'/exp OR 'middle aged'/exp OR aged:ti OR adult*:ti OR older:ti 5,151,469

#05. 'middle aged':ti,ab OR ageing:ti,ab OR aging:ti,ab OR elderly:ti,ab OR 'old age':ti,ab OR elder:ti,ab OR senior*:ti,ab OR 'older adult*':ti,ab OR 'over 45':ti,ab OR 'over 50':ti,ab OR 'over 55':ti,ab OR 'over 60':ti,ab OR 'over 65':ti,ab OR 'over 70':ti,ab OR 'over 75':ti,ab OR 'over 80':ti,ab OR 'over 85':ti,ab OR '45 and over':ti,ab OR '50 and over':ti,ab OR '55 and over':ti,ab OR '60 and over':ti,ab OR '65 and over':ti,ab OR '70 and over':ti,ab OR '75 and over':ti,ab OR '80 and over':ti,ab OR '85 and over':ti,ab OR geriatric:ti,ab OR quinquagenarian*:ti,ab OR sexagenarian*:ti,ab OR septuagenarian*:ti,ab OR octogenarian*:ti,ab OR nonagenarian*:ti,ab 1,029,445

#06. #4 OR #5 5,587,099

#07. 'healthy aging'/exp OR 'healthy aging':ti,ab OR 'healthy ageing':ti,ab OR 'ageing well':ti,ab OR 'aging well':ti,ab OR 'well ageing':ti,ab OR 'well aging':ti,ab OR 'ageing in place':ti,ab OR 'aging in place':ti,ab OR 'successful ageing':ti,ab OR 'successful aging':ti,ab OR 'healthy living':ti,ab OR 'health indicator*':ti,ab OR 'health promotion':ti,ab 62,027

#08. healthy:ti OR policy:ti OR policies:ti OR prevention*:ti OR guideline*:ti OR legislation*:ti OR act:ti OR model:ti OR law:ti OR laws:ti OR promotion:ti OR promoting:ti OR intervention*:ti 1,470,889

#09. #7 OR #8 1,510,301

#10. #3 AND #6 AND #9 1,189

#11. ('animal'/exp OR 'invertebrate'/exp OR 'animal experiment'/de OR 'animal model'/de OR 'animal tissue'/de OR 'animal cell'/de OR 'nonhuman'/de) AND ('human'/de OR 'normal human'/de) 24,808,248

#12. 'animal'/exp OR 'invertebrate'/exp OR 'animal experiment'/de OR 'animal model'/de OR 'animal tissue'/de OR 'animal cell'/de OR 'nonhuman'/de 32,335,574

#13. #12 NOT #11 7,527,326

#14. #10 NOT #13 1,038

#15. #14 NOT (child:ti OR children:ti OR infant*:ti OR 'young adult*':ti OR mouse:ti OR mice:ti OR murine:ti OR genetic:ti OR childhood:ti OR tissue*:ti OR rat:ti OR rats:ti OR prenatal:ti OR antenatal:ti) 912

#16. #14 NOT (child:ti OR children:ti OR infant*:ti OR 'young adult*':ti OR mouse:ti OR mice:ti OR murine:ti OR genetic:ti OR childhood:ti OR tissue*:ti OR rat:ti OR rats:ti OR prenatal:ti OR antenatal:ti) AND [embase]/lim 660

#17. #14 NOT (child:ti OR children:ti OR infant*:ti OR 'young adult*':ti OR mouse:ti OR mice:ti OR murine:ti OR genetic:ti OR childhood:ti OR tissue*:ti OR rat:ti OR rats:ti OR prenatal:ti OR antenatal:ti) AND [medline]/lim 641

#18. #16 NOT #17 270

#19. #18 AND ([english]/lim) 260

Total number of records from databases: 6,932

Total number of unique records after removal of duplicates in EndNote and Covidence: 3,443

UPDATE SEARCH “ID AND HEALTHY AGING”

PROJECT INFORMATION

REQUEST DATE	NAME	INSTITUTION/ORGANISATION
2024-02-14	Gerd Ahlström, Nadia El Mayyran	Department of Health Sciences

SEARCH STRATEGIES

This search strategy documentation is the same as from 2022-04-15.

Databases searched:

Academic Search Complete, APA PsycInfo, Cinahl Complete, Cochrane Library, Embase, ERIC, PubMed, Scopus, SocIndex, Urban Studies Abstracts, Web of Science Core Collection

PubMed (National Library of Medicine)
Date of search: 2024-02-14

#1 ("Intellectual Disability"[Mesh] OR "intellectual disabilit*" OR "Down Syndrome"[Mesh] OR "Down Syndrome" OR "intellectual abnormalit*" OR "learning abnormalit*" OR "mental abnormalit*" OR “neurodevelopmental abnormali*” OR "intellectual deficienc*" OR "learning deficienc*" OR "mental deficienc*" OR “neurodevelopmental deficienc*” OR "mental retardation" OR "developmental disabilit*" OR "learning disabilit*") 182,610

#2 (aged[MeSH] OR Aged[TI] OR adult*[TI] OR older[TI] OR "middle aged"[Mesh] OR “middle aged”[TW] OR ageing[TW] OR aging[TW] OR elderly[TW] OR “old age”[TW] OR elder[TW] OR senior*[TW] OR “older adult*”[TW] OR “over 45” [TW] OR "over 50"[TW] OR "over 55“[TW] OR "over 60"[TW] OR "over 65"[TW] OR “over 70” [TW] OR “over 75” [TW] OR "over 80"[TW] OR “over 85” [TW] OR "45 and over"[TW] OR "50 and over"[TW] OR "55 and over"[TW] OR "60 and over"[TW] OR "65 and over"[TW] OR "70 and over"[TW] OR "75 and over"[TW] OR "80 and over"[TW] OR "85 and over"[TW] OR geriatric[TW] OR quinquagenarian*[TW] OR sexagenarian*[TW] OR septuagenarian*[TW] OR octogenarian*[TW] OR nonagenarian*[TW]) 6,381,202

#3 ("Healthy Aging"[Mesh] OR "healthy aging" OR "healthy ageing" OR "ageing well" OR "aging well" OR "well ageing" OR "well aging" OR “ageing in place” OR “aging in place” OR "successful ageing" OR "successful aging" OR "healthy living" OR "health indicator*" OR “health promotion” OR healthy[TI] OR policy[TI] OR policies[TI] OR prevention*[TI] OR intervention*[TI] OR guideline*[TI] OR legislation*[TI] OR act[TI] OR model[TI] OR law[TI] OR laws[TI] OR promotion[TI] OR promoting[TI]) 1,434,772

#4 #1 AND #2 AND #3 1,397

#5 #4 NOT (child[TI] OR children[TI] OR infant*[TI] OR “young adult*”[TI] OR mouse[TI] OR mice[TI] OR murine[TI] OR genetic[TI] OR childhood[TI] OR tissue*[TI] OR rat[TI] OR rats[TI] OR prenatal[TI] OR antenatal[TI]) 1,075

#6 #5 NOT (animals[MH] NOT humans[MH]) 1,052

Filters: English 1,021

Filter: (("2022/04/01"[Date - Publication] : "2023/12/31"[Date - Publication])) 115

The search filter for older/elderly people was adjusted from the Canadian Health Libraries Association, https://extranet.santecom.qc.ca/wiki/!biblio3s/doku.php?id=concepts:personnes_agees

CINAHLComplete (Cumulative Index to Nursing and Allied Health Literature; EbscoHost, inception to present)

Date of search: 2024-02-14

S1	((MH "Intellectual Disability+") OR (MH "Down Syndrome")) OR ("intellectual disabilit*" OR "Down Syndrome" OR "intellectual abnormalit*" OR "learning abnormalit*" OR "mental abnormalit*") OR (“neurodevelopmental abnormali*” OR "intellectual deficienc*" OR "learning deficienc*" OR "mental deficienc*" OR “neurodevelopmental deficienc*” OR "mental retardation" OR "developmental disabilit*" OR "learning disabilit*")	63,912
S2	((MH "Aged+") OR (MH "Middle Age")) OR TI (aged OR adult* OR older) OR ("middle aged" OR ageing OR aging OR elderly OR "old age" OR elder OR senior* OR "older adult*" OR "over 45" OR "over 50" OR "over 55") OR ("over 60" OR "over 65" OR "over 70" OR "over 75" OR "over 80" OR "over 85" OR "45 and over" OR "50 and over" OR "55 and over" OR "60 and over" OR "65 and over" OR "70 and over" OR "75 and over" OR "80 and over" OR "85 and over") OR (geriatric OR quinquagenarian* OR sexagenarian* OR septuagenarian* OR octogenarian* OR nonagenarian*)	1,732,082
S3	((MH "Healthy Aging") OR "healthy aging" OR "healthy ageing" OR "ageing well" OR "aging well" OR "well ageing" OR "well aging" OR “ageing in place” OR “aging in place”) OR ("successful ageing" OR "successful aging" OR "healthy living" OR "health indicator*" OR “health promotion”) OR TI (healthy OR policy OR policies OR prevention* OR guideline* OR legislation* OR act OR model OR law OR laws OR promotion OR promoting OR intervention*)	579,963
S4	S1 AND S2 AND S3	937
S5	(S1 AND S2 AND S3) NOT TI (child OR children OR infant* OR "young adult*" OR mouse OR mice OR murine OR genetic OR childhood OR tissue* OR rat OR rats OR prenatal OR antenatal)	820
S6	Filter: English	725
S7	Publication Date: 20220401-20231231	81

APA PsycInfo (EbscoHost, inception to present)
SocIndex (EbscoHost, inception to present)
Date of search: 2024-02-14

S1	((DE "Learning Disabilities") OR (DE "MENTAL disabilities")) OR ("intellectual disabilit*" OR "Down Syndrome" OR "intellectual abnormalit*" OR "learning abnormalit*" OR "mental abnormalit*") OR (“neurodevelopmental abnormali*” OR "intellectual deficienc*" OR "learning deficienc*" OR "mental deficienc*" OR “neurodevelopmental deficienc*” OR "mental retardation" OR "developmental disabilit*" OR "learning disabilit*")	22,794
S2	(DE "OLDER people") OR (DE "MIDDLE-aged persons" OR DE "MIDDLE age" OR DE "OLD age") OR TI (aged OR adult* OR older) OR ("middle aged" OR ageing OR aging OR elderly OR "old age" OR elder OR senior* OR "older adult*" OR "over 45" OR "over 50" OR "over 55") OR ("over 60" OR "over 65" OR "over 70" OR "over 75" OR "over 80" OR "over 85" OR "45 and over" OR "50 and over" OR "55 and over" OR "60 and over" OR "65 and over" OR "70 and over" OR "75 and over" OR "80 and over" OR "85 and over") OR (geriatric OR quinquagenarian* OR sexagenarian* OR septuagenarian* OR octogenarian* OR nonagenarian*)	167,892
S3	(DE "ACTIVE aging" OR DE "SUCCESSFUL aging") OR ("healthy aging" OR "healthy ageing" OR "ageing well" OR "aging well" OR "well ageing" OR "well aging" OR “ageing in place” OR “aging in place”) OR ("successful ageing" OR "successful aging" OR "healthy living" OR "health indicator*" OR “health promotion”) OR TI (healthy OR policy OR policies OR prevention* OR guideline* OR legislation* OR act OR model OR law OR laws OR promotion OR promoting OR intervention*)	210,305
S4	S1 AND S2 AND S3	161
S5	(S1 AND S2 AND S3) NOT TI (child OR children OR infant* OR "young adult*" OR mouse OR mice OR murine OR genetic OR childhood OR tissue* OR rat OR rats OR prenatal OR antenatal)	151
S6	Filter: English	151
S7	Publication Date: 20220401-20231231	12

Urban Studies Abstracts (EbscoHost, inception to present)
Date of search: 2024-02-14

#	Query	Results
S1	("intellectual disabilit*" OR "Down Syndrome" OR "intellectual abnormalit*" OR "learning abnormalit*" OR "mental abnormalit*") OR (“neurodevelopmental abnormali*” OR "intellectual deficienc*" OR "learning deficienc*" OR "mental deficienc*" OR “neurodevelopmental deficienc*” OR "mental retardation" OR "developmental disabilit*" OR "learning disabilit*")	137
S2	TI (aged OR adult* OR older) OR ("middle aged" OR ageing OR aging OR elderly OR "old age" OR elder OR senior* OR "older adult*" OR "over 45" OR "over 50" OR "over 55") OR ("over 60" OR "over 65" OR "over 70" OR "over 75" OR "over 80" OR "over 85" OR "45 and over" OR "50 and over" OR "55 and over" OR "60 and over" OR "65 and over" OR "70 and over" OR "75 and over" OR	3,744

	"80 and over" OR "85 and over") OR (geriatric OR quinquagenarian* OR sexagenarian* OR septuagenarian* OR octogenarian* OR nonagenarian*)	
S3	(("healthy aging" OR "healthy ageing" OR "ageing well" OR "aging well" OR "well ageing" OR "well aging" OR “ageing in place” OR “aging in place”) OR ("successful ageing" OR "successful aging" OR "healthy living" OR "health indicator*" OR “health promotion”) OR TI (healthy OR policy OR policies OR prevention* OR guideline* OR legislation* OR act OR model OR law OR laws OR promotion OR promoting OR intervention*)	13,580
S4	S1 AND S2 AND S3	2
S5	(S1 AND S2 AND S3) NOT TI (child OR children OR infant* OR "young adult*" OR mouse OR mice OR murine OR genetic OR childhood OR tissue* OR rat OR rats OR prenatal OR antenatal)	2
S6	Publication Date: 20220401-20231231	0

ERIC (EbscoHost, inception to present)
Date of search: 2024-02-14

S1	(DE "Intellectual Disability" OR DE "Down Syndrome" OR DE "Mild Intellectual Disability" OR DE "Moderate Intellectual Disability" OR DE "Severe Intellectual Disability") OR ("intellectual disabilit*" OR "Down Syndrome" OR "intellectual abnormalit*" OR "learning abnormalit*" OR "mental abnormalit*") OR (“neurodevelopmental abnormali*” OR "intellectual deficienc*" OR "learning deficienc*" OR "mental deficienc*" OR “neurodevelopmental deficienc*” OR "mental retardation" OR "developmental disabilit*" OR "learning disabilit*")	56,047
S2	(DE "Older Adults”) OR TI (aged OR adult* OR older) OR ("middle aged" OR ageing OR aging OR elderly OR "old age" OR elder OR senior* OR "older adult*" OR "over 45" OR "over 50" OR "over 55") OR ("over 60" OR "over 65" OR "over 70" OR "over 75" OR "over 80" OR "over 85" OR "45 and over" OR "50 and over" OR "55 and over" OR "60 and over" OR "65 and over" OR "70 and over" OR "75 and over" OR "80 and over" OR "85 and over") OR (geriatric OR quinquagenarian* OR sexagenarian* OR septuagenarian* OR octogenarian* OR nonagenarian*)	85,408
S3	("healthy aging" OR "healthy ageing" OR "ageing well" OR "aging well" OR "well ageing" OR "well aging" OR “ageing in place” OR “aging in place”) OR ("successful ageing" OR "successful aging" OR "healthy living" OR "health indicator*" OR “health promotion”) OR TI (healthy OR policy OR policies OR prevention* OR guideline* OR legislation* OR act OR model OR law OR laws OR promotion OR promoting OR intervention*)	139,365
S4	S1 AND S2 AND S3	353
S5	(S1 AND S2 AND S3) NOT TI (child OR children OR infant* OR "young adult*" OR mouse OR mice OR murine OR genetic OR childhood OR tissue* OR rat OR rats OR prenatal OR antenatal)	298
S6	Filter: English	265
S7	Publication Date: 20220401-20231231	20

Academic Search Complete (EbscoHost, inception to present)

Date of search: 2024-02-14

S1	(DE "MENTAL disabilities") OR (DE "DOWN syndrome") OR ("intellectual disabilit*" OR "Down Syndrome" OR "intellectual abnormalit*" OR "learning abnormalit*" OR "mental abnormalit*") OR (“neurodevelopmental abnormali*” OR "intellectual deficienc*" OR "learning deficienc*" OR "mental deficienc*" OR “neurodevelopmental deficienc*”) OR ("mental retardation" OR "developmental disabilit*" OR "learning disabilit*")	123,703
S2	(DE "OLDER people" OR DE "MIDDLE age" OR DE "MIDDLE-aged persons" OR DE "OLD age") OR (TI (aged OR adult* OR older)) OR ("middle aged" OR ageing OR aging OR elderly OR "old age" OR elder OR senior* OR "older adult*" OR "over 45" OR "over 50" OR "over 55")) OR ("over 60" OR "over 65" OR "over 70" OR "over 75" OR "over 80" OR "over 85" OR "45 and over" OR "50 and over" OR "55 and over" OR "60 and over" OR "65 and over" OR "70 and over" OR "75 and over" OR "80 and over" OR "85 and over") OR (geriatric OR quinquagenarian* OR sexagenarian* OR septuagenarian* OR octogenarian* OR nonagenarian*)	1,306,026
S3	DE "ACTIVE aging" OR ("healthy aging" OR "healthy ageing" OR "ageing well" OR "aging well" OR "well ageing" OR "well aging" OR “ageing in place” OR “aging in place”) OR ("successful ageing" OR "successful aging" OR "healthy living" OR "health indicator*" OR “health promotion”) OR TI (healthy OR policy OR policies OR prevention* OR guideline* OR legislation* OR act OR model OR law OR laws OR promotion OR promoting OR intervention*)	1,695,321
S4	S1 AND S2 AND S3	1,303
S5	(S1 AND S2 AND S3) NOT TI (child OR children OR infant* OR "young adult*" OR mouse OR mice OR murine OR genetic OR childhood OR tissue* OR rat OR rats OR prenatal OR antenatal)	1,047
S6	Filter: English	1,035
S7	Publication Date: 20220401-20231231	103

Scopus

Date of search: 2024-02-14

#1 TITLE-ABS-KEY("intellectual disabilit*" OR "down syndrome" OR "intellectual abnormalit*" OR "learning abnormalit*" OR "mental abnormalit*" OR "neurodevelopmental abnormali*" OR "intellectual deficienc*" OR "learning deficienc*" OR "mental deficienc*" OR "neurodevelopmental deficienc*" OR "mental retardation" OR "developmental disabilit*" OR "learning disabilit*") 214,491

#2 (TITLE(aged OR adult* OR older) OR TITLE-ABS-KEY("middle aged" OR ageing OR aging OR elderly OR "old age" OR elder OR senior* OR "older adult*" OR "over 45" OR "over 50" OR "over 55" OR "over 60" OR "over 65" OR "over 70" OR "over 75" OR "over 80" OR "over 85") OR TITLE-ABS-KEY("45 and over" OR "50 and over" OR "55 and over" OR "60 and over" OR "65 and over" OR "70 and over" OR "75 and over" OR "80 and over" OR "85 and over") OR TITLE-ABS-KEY(geriatric OR quinquagenarian* OR sexagenarian* OR septuagenarian* OR octogenarian* OR nonagenarian*)) 5,983,789

#3 (TITLE-ABS-KEY("healthy aging" OR "healthy ageing" OR "ageing well" OR "aging well" OR "well ageing" OR "well aging" OR "ageing in place" OR "aging in place") OR TITLE-ABS-KEY("successful ageing" OR "successful aging" OR "healthy living" OR "health indicator*" OR "health promotion") OR TITLE(healthy OR policy OR policies

OR prevention* OR intervention* OR guideline* OR legislation* OR act OR model OR law OR laws OR promotion OR promoting)) 4,381,069

#4 #1 AND #2 AND #3 1,743

#5 TITLE(child OR children OR infant* OR "young adult*" OR mouse OR mice OR murine OR genetic OR childhood OR tissue* OR rat OR rats OR prenatal OR antenatal) 4,175,384

#6 #4 AND NOT #5 1,357

#7 Filters: English 1,304

#8 Publication year 2022-2023 148

Cochrane Library via Cochrane Library Online (Wiley, Issue 2 of 12, February 2024)

Date of search: 2024-02-14

#1 MeSH descriptor: [Intellectual Disability] explode all trees 1,999

#2 MeSH descriptor: [Down Syndrome] explode all trees 520

#3 ("intellectual disability" OR "Down Syndrome" OR "intellectual abnormality" OR "learning abnormality" OR "mental abnormality" OR “neurodevelopmental abnormality” OR "intellectual deficiency" OR "learning deficiency" OR "mental deficiency" OR “neurodevelopmental deficiency” OR "mental retardation" OR "developmental disability" OR "learning disability"):ti,ab,kw (Word variations have been searched) 5,957

#4 #1 OR #2 OR #3 6,353

#5 MeSH descriptor: [Aged] explode all trees 273,647

#6 MeSH descriptor: [Middle Aged] explode all trees 404,324

#7 (Aged OR adult* OR older):ti (Word variations have been searched) 117,424

#8 ("middle aged" OR ageing OR aging OR elderly OR "old age" OR elder OR senior* OR "older adults" OR "over 45" OR "over 50" OR "over 55"):ti,ab,kw OR ("over 60" OR "over 65" OR "over 70" OR "over 75" OR "over 80" OR "over 85" OR "45 and over" OR "50 and over" OR "55 and over" OR "60 and over"):ti,ab,kw OR ("65 and over" OR "70 and over" OR "75 and over" OR "80 and over" OR "85 and over"):ti,ab,kw OR (geriatric OR quinquagenarian* OR sexagenarian* OR septuagenarian* OR octogenarian* OR nonagenarian*):ti,ab,kw (Word variations have been searched) 930,967

#9 #5 OR #6 OR #7 OR #8 955,899

#10 MeSH descriptor: [Healthy Aging] explode all trees 118

#11 ("healthy aging" OR "healthy ageing" OR "ageing well" OR "aging well" OR "well ageing" OR "well aging" OR “ageing in place” OR “aging in place” OR "successful ageing" OR "successful aging" OR "healthy living" OR "health indicator*" OR “health promotion”):ti,ab,kw (Word variations have been searched) 15,881

#12 (healthy OR policy OR policies OR prevention* OR guideline* OR legislation* OR act OR model OR law OR laws OR promotion OR promoting OR intervention*):ti (Word variations have been searched) 205,735

#13 #10 OR #11 OR #12 213,095

#14 #4 AND #9 AND #13 523

#15 (child OR children OR infant* OR "young adults" OR mouse OR mice OR murine OR genetic OR childhood OR tissue* OR rat OR rats OR prenatal OR antenatal):ti,ab,kw (Word variations have been searched) 485,278

#16 #14 NOT #15 150

#17 Year first published 2022-2023 23

Web of Science Core collection (Clarivate Analytics)

Date of search: 2024-02-14

Databases searched in Web of Science Core Collection (inception as described below)

Science Citation Index Expanded (SCI-EXPANDED) --1900-present

Social Sciences Citation Index (SSCI) --1956-present

Arts & Humanities Citation Index (A&HCI) --1975-present

#1 "intellectual disabilit*" OR "down syndrome" OR "intellectual abnormalit*" OR "learning abnormalit*" OR "mental abnormalit*" OR "neurodevelopmental abnormali*" OR "intellectual deficienc*" OR "learning deficienc*" OR "mental deficienc*" OR "neurodevelopmental deficienc*" OR "mental retardation" OR "developmental disabilit*" OR "learning disabilit*" (Topic) 116,931

#2 aged OR adult* OR older (Title) or "middle aged" OR ageing OR aging OR elderly OR "old age" OR elder OR senior* OR "older adult*" OR "over 45" OR "over 50" OR "over 55" OR "over 60" OR "over 65" OR "over 70" OR "over 75" OR "over 80" OR "over 85" (Topic) or "45 and over" OR "50 and over" OR "55 and over" OR "60 and over" OR "65 and over" OR "70 and over" OR "75 and over" OR "80 and over" OR "85 and over" (Topic) or geriatric OR quinquagenarian* OR sexagenarian* OR septuagenarian* OR octogenarian* OR nonagenarian* (Topic) 5,468,113

#3 "healthy aging" OR "healthy ageing" OR "ageing well" OR "aging well" OR "well ageing" OR "well aging" OR "ageing in place" OR "aging in place" (Topic) or "successful ageing" OR "successful aging" OR "healthy living" OR "health indicator*" OR "health promotion" (Topic) or healthy OR policy OR policies OR prevention* OR intervention* OR guideline* OR legislation* OR act OR model OR law OR laws OR promotion OR promoting (Title) 3,927,068

#4 #1 AND #2 AND #3 2,232

#5 #4 NOT TI=(child OR children OR infant* OR "young adult*" OR mouse OR mice OR murine OR genetic OR childhood OR tissue* OR rat OR rats OR prenatal OR antenatal) 1,578

#6 Limit English 1,560

#7 Publication years 2022-2023 195

Embase.com (Elsevier, 1947-present)

2024-02-14

#01. 'mental deficiency'/exp OR 'down syndrome'/exp 168,829

#02. 'intellectual disabilit*':ti,ab OR 'down syndrome':ti,ab OR 'intellectual abnormalit*':ti,ab OR 'learning abnormalit*':ti,ab OR 'mental abnormalit*':ti,ab OR 'neurodevelopmental abnormalit*':ti,ab OR 'intellectual deficienc*':ti,ab OR 'learning deficienc*':ti,ab OR 'mental deficienc*':ti,ab OR 'neurodevelopmental deficienc*':ti,ab OR 'mental retardation':ti,ab OR 'developmental disabilit*':ti,ab OR 'learning disabilit*':ti,ab 114,395

#03. #1 OR #2 227,528

#04. 'aged'/exp OR 'middle aged'/exp OR aged:ti OR adult*:ti OR older:ti 5,765,661

#05. 'middle aged':ti,ab OR ageing:ti,ab OR aging:ti,ab OR elderly:ti,ab OR 'old age':ti,ab OR elder:ti,ab OR senior*:ti,ab OR 'older adult*':ti,ab OR 'over 45':ti,ab OR 'over 50':ti,ab OR 'over 55':ti,ab OR 'over 60':ti,ab OR 'over 65':ti,ab OR 'over 70':ti,ab OR 'over 75':ti,ab OR 'over 80':ti,ab OR 'over 85':ti,ab OR '45 and over':ti,ab OR '50 and over':ti,ab OR '55 and over':ti,ab OR '60 and over':ti,ab OR '65 and over':ti,ab OR '70 and over':ti,ab OR

'75 and over':ti,ab OR '80 and over':ti,ab OR '85 and over':ti,ab OR geriatric:ti,ab OR quinquagenarian*:ti,ab OR sexagenarian*:ti,ab OR septuagenarian*:ti,ab OR octogenarian*:ti,ab OR nonagenarian*:ti,ab 1,154,145

#06. #4 OR #5 6,250,311

#07. 'healthy aging'/exp OR 'healthy aging':ti,ab OR 'healthy ageing':ti,ab OR 'ageing well':ti,ab OR 'aging well':ti,ab OR 'well ageing':ti,ab OR 'well aging':ti,ab OR 'ageing in place':ti,ab OR 'aging in place':ti,ab OR 'successful ageing':ti,ab OR 'successful aging':ti,ab OR 'healthy living':ti,ab OR 'health indicator*':ti,ab OR 'health promotion':ti,ab 71,002

#08. healthy:ti OR policy:ti OR policies:ti OR prevention*:ti OR guideline*:ti OR legislation*:ti OR act:ti OR model:ti OR law:ti OR laws:ti OR promotion:ti OR promoting:ti OR intervention*:ti 1,639,467

#09. #7 OR #8 1,685,121

#10. #3 AND #6 AND #9 1,347

#11. ('animal'/exp OR 'invertebrate'/exp OR 'animal experiment'/de OR 'animal model'/de OR 'animal tissue'/de OR 'animal cell'/de OR 'nonhuman'/de) AND ('human'/de OR 'normal human'/de) 27,228,075

#12. 'animal'/exp OR 'invertebrate'/exp OR 'animal experiment'/de OR 'animal model'/de OR 'animal tissue'/de OR 'animal cell'/de OR 'nonhuman'/de 35,191,042

#13. #12 NOT #11 7,962,967

#14. #10 NOT #13 1,176

#15. #14 NOT (child:ti OR children:ti OR infant*:ti OR 'young adult*':ti OR mouse:ti OR mice:ti OR murine:ti OR genetic:ti OR childhood:ti OR tissue*:ti OR rat:ti OR rats:ti OR prenatal:ti OR antenatal:ti) 1,028

#16. #14 NOT (child:ti OR children:ti OR infant*:ti OR 'young adult*':ti OR mouse:ti OR mice:ti OR murine:ti OR genetic:ti OR childhood:ti OR tissue*:ti OR rat:ti OR rats:ti OR prenatal:ti OR antenatal:ti) AND [embase]/lim 752

#17. #14 NOT (child:ti OR children:ti OR infant*:ti OR 'young adult*':ti OR mouse:ti OR mice:ti OR murine:ti OR genetic:ti OR childhood:ti OR tissue*:ti OR rat:ti OR rats:ti OR prenatal:ti OR antenatal:ti) AND [medline]/lim 710

#18. #16 NOT #17 311

#19. #18 AND ([english]/lim) 301

#20. #19 AND [2022-2023]/py 38

Summary of update searches (2024-02-14)

Total number of records from all databases:	794
Number after automatic deduplication in EndNote:	469
Number after automatic deduplication in Covidence:	335
